# Improving Internal Medicine Resident Comfort With Shoulder and Knee Joint Injections Using an Injection Workshop

**DOI:** 10.15766/mep_2374-8265.10979

**Published:** 2020-09-28

**Authors:** Michael K. Seifert, Christina T. Holt, Amy Haskins, William Dexter

**Affiliations:** 1 Assistant Professor, Department of Orthopedics, University of North Carolina at Chapel Hill School of Medicine; 2 Assistant Professor, Department of Family Medicine, Maine Medical Center; 3 Professor, Department of Family Medicine, Maine Medical Center and Tufts University School of Medicine

**Keywords:** Joint Injections, Resident Teaching, Sports Medicine, Internal Medicine, Clinical/Procedural Skills Training

## Abstract

**Introduction:**

Joint injections can be effective treatments for musculoskeletal issues. We examined whether a brief teaching session delivered to residents and faculty would significantly improve resident confidence in performing shoulder and knee joint injections.

**Methods:**

We implemented a 90-minute workshop instructed by two sports medicine providers. The objectives and content of the workshop included the topics of indications and contraindications, risks and benefits, supplies and setup, and injection techniques, all assessed on 5-point Likert scales. The workshop included a lecture, followed by residents practicing injections on simulation models and identifying key bony landmarks. Outpatient clinic faculty were given the same lecture and practiced on models. The postworkshop questionnaire was administered to the residents 4 months later.

**Results:**

Eighteen residents participated. Mean confidence for performing knee injections increased from 2.2 to 3.8 immediately postlecture (*p* = .006). Shoulder injection confidence increased from 1.6 to 3.8 immediately postlecture (*p* = .0002). Confidence in knowledge of the risks and benefits, supplies needed, and indications increased similarly. Four months postworkshop, confidence levels were sustained above pretesting levels for all areas studied. Faculty members appreciated their workshop since they had not often performed injections.

**Discussion:**

This brief workshop-style teaching session can provide meaningful, durable improvements in a trainee's confidence regarding performing shoulder or knee joint injections. The session requires few resources and fits into regular didactic sessions. Further development of this model could increase clinical performance and practice confidence and make these procedures more widely accessible to patients.

## Educational Objectives

By the end of this activity, participants will be able to:

1. Report higher confidence in their ability to perform a subacromial shoulder injection.
2. Report higher confidence in their ability to perform an intra-articular knee injection.
3. Describe the indications, risks, and benefits of a subacromial shoulder or knee injection.
4. List supplies needed for a subacromial shoulder or knee injection.

## Introduction

Musculoskeletal issues prompt 10%-15% of primary care visits.^1^ Joint injections can be an effective treatment for conditions such as osteoarthritis and tendonitis.^2,3^ Studies show that internal medicine (IM) residents lack confidence in performing joint injections,^4–6^ citing lack of training.^4^ Resident confidence levels for knee and shoulder injections were found to be as low as 10%. There is evidence that academic physicians (specifically IM) perform and precept few joint injections,^6^ but participation in dedicated workshops and curricula can improve confidence of trainees performing these procedures.^7–12^

Previous studies have focused training sessions lasting half a day or longer or using a cadaver lab. These types of programs may be difficult to implement given other training needs, supervising physician schedules, and room or other resource availability. Additionally, many previous musculoskeletal workshops have provided training on a large number of joints, rather than focusing on those most commonly seen in ambulatory care and thus most clinically useful. In hopes of creating a more easily adaptable program, we aimed to determine if a brief landmark-based teaching session delivered to both residents and faculty would significantly improve IM residents' confidence in their ability to perform joint injections.

Expanding on a pilot workshop, designed for residents but performed at a different institution,^13^ we developed two new workshops: one aimed at IM residents and one for the IM supervising faculty. We hypothesized that this teaching intervention, delivered early in the training year, would improve resident self-confidence in knowledge about and ability to perform joint injections immediately postintervention and at 4-month follow-up.

## Methods

We implemented a 90-minute resident joint injection workshop in September 2016, scheduled during the usual residency didactic time, in a large conference room. Two sports medicine–trained providers offered instruction using a predesigned teaching plan (Appendix A). The residents were not expected to have any prerequisite knowledge outside of the anatomical terms and basic information about standard medications used in joint injections, namely, lidocaine and corticosteroids. The training goals of the workshop included the topics of indications and contraindications, risks and benefits, supplies and setup, anatomy, external landmarks, and injection techniques. These goals were derived from previous studies.^14–18^

The workshop included three interactive components: a lecture, drawing of bony landmarks, and practice with joint injection models. The shoulder models were from Limbs and Things, and knee models were from Limbs and Things and Simulab. The models had realistic skin texture with underlying palpable bony anatomy. They were placed on standard tables at the side of educational room with space for three to four residents around the model. Additional supplies included a computer with access to PowerPoint, a projector, skin markers, and empty syringes with attached injection needles.

A preworkshop questionnaire (Appendix D) measured residents' self-reported confidence with clinical indications, supplies, techniques, and risks and benefits (using 5-point Likert scales: 1 = *not confident,* 5 = *very confident*). Each of the following items was presented for both intra-articular knee injections and subacromial shoulder injections: “I recognize the clinical indications,” “I can explain the risks and benefits to my patients,” “I know the supplies I would need to perform,” and “I can perform safely and accurately.”

The primary author led a 40-minute lecture, using a PowerPoint presentation (Appendix B), to review the above-mentioned content areas, with key points provided as a handout (Appendix C) to each attendee. Subsequently, some groups of three to four residents practiced injections on simulation models that had been set up at the side of room, on a table, prior to the start of the lecture. In rotations, every resident practiced performing at least two injections on each joint model and observed their small-group partners. Simultaneously, other groups spent time identifying key bony landmarks, as highlighted in the lecture, and marking them on each other's skin using skin markers. Workgroups rotated every 15 minutes. The two instructors rotated between the joint models and landmark drawing to supervise and answer questions. Following the workshop, the questionnaire was administered again (Appendix E) with the additional item “I am more likely to perform an injection with my clinic patients if indicated” (1 = *much less likely,* 5 = *much more likely*).

The following month, eight IM outpatient clinic faculty were given the same lecture and opportunity to practice on models at a scheduled faculty meeting led and supervised by the primary author. The faculty lecture included a handout, but there was no station or time allotted for drawing bony landmarks. No pre- or postworkshop questionnaire was given to the faculty.

The postworkshop questionnaire was administered to the residents 4 months later online (Appendix F), using SurveyMonkey, to assess the durability of any confidence gains after the teaching intervention. We compared pre- and immediate postintervention paired scores for each question using Wilcoxon signed rank tests and used Mann-Whitney *U* tests to compare (unpaired) preintervention scores to the 4-month postintervention scores.

## Results

**Figure 1.**
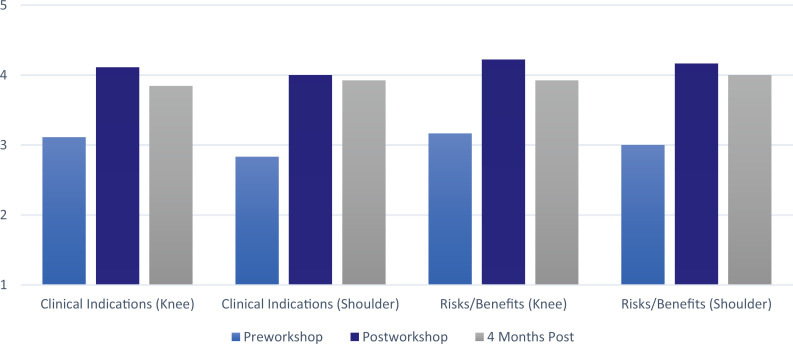
Sustained improvement in resident confidence of knowledge of clinical indications and risks/benefits of knee and shoulder injections, *p* ≤ .05.

**Figure 2.**
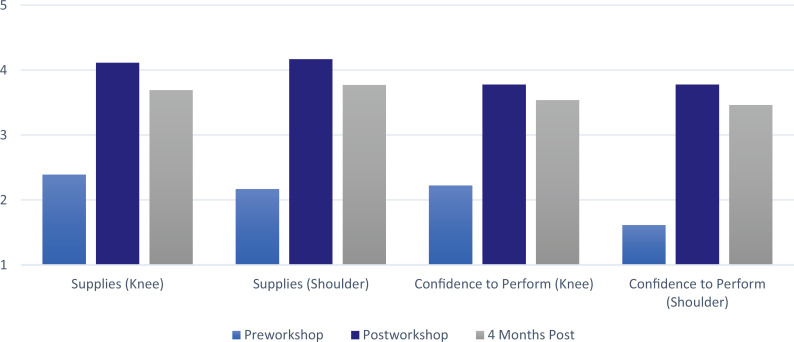
Sustained improvement in resident confidence of knowledge of supplies needed and ability to perform knee and shoulder injections, *p* ≤ .05.

Eighteen IM residents attended and completed the workshop (out of 45 eligible; 40% participation). All participants completed the pre- and postworkshop questionnaires. At the 4-month follow-up, 13 out of 18 participants completed the questionnaire. Confidence for performing knee injections increased from a mean of 2.2 to a mean of 3.8 immediately postworkshop (*p* = .006). The mean improvement in confidence 4 months postworkshop was maintained at 3.5 (*p* = .006). For shoulder injections, the mean confidence in performing increased from 1.6 to 3.8 pre- to postworkshop (*p* = .0002) and was 3.5 at 4 months (*p* < .0001). Confidence in knowledge of the risks and benefits, supplies needed, and indications increased similarly (Figures 1 and 2). Paired *t* tests of all measured items showed statistically significant increases immediately postintervention. Four months postworkshop, confidence levels were sustained above the pretesting levels for all areas studied (*p* ≤ .05, *n* = 13; see Table). Finally, residents indicated they were more likely to perform both knee and shoulder injections, with means of 4.4 and 4.3 immediately postworkshop and 3.8 and 3.9 at 4 months for the knee and shoulder (*p* = .009 and .01), respectively.

**Table.**
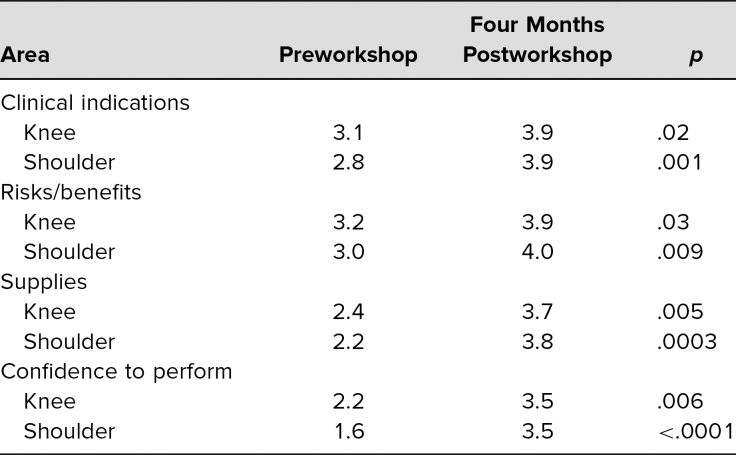
Statistically Significant Gains in Confidence in All Areas Studied

## Discussion

We believe that this brief, workshop-style teaching session can provide meaningful, durable improvements in a trainee's confidence of performing a shoulder or knee joint injection. This type of teaching session requires few resources and can fit into the regular didactic sessions of a training program. Other studies have used longer or resource-intensive sessions and included additional joints.^7–12^ We believe that the focus should be on the most common injections performed by primary care providers. We also believe that a brief session for clinic faculty is beneficial, since they supervise trainees performing these procedures in their outpatient clinics. Faculty members commented that they appreciated the workshop since they had not performed injections frequently in the recent past. Further work is needed to see if the faculty workshop leads faculty to supervise more trainee-performed joint injections.

Residencies across the country are using workshop techniques for procedural skill training, including with central line placement, endotracheal tube placement, and Advanced Cardiovascular Life Support code practice.^19,20^ Joint injections training lends itself well to this model. The trainees have the opportunity to work with supplies, incorrect techniques, and forgotten anatomy in a setting where they can pause and ask questions among peers.

The participation rate in our resident teaching session was limited to 18 out of 45 residents. This could reflect self-selection of attendees, which might increase attention and learning. The 4-month follow-up survey had a good response rate indicating sustained learning. Increased exposure to joint injections during the intervening 4 months of training could also have led to increased joint injection confidence, even without our workshop. Improving resident attendance and conducting the faculty training with landmarks might be changes for the future. Additionally, we could survey the faculty for their confidence and changes before and after the training.

This project did not survey our residents regarding the effect of the preceptor training or actual injections performed in clinical settings, but we would add this to future iterations. To address the initial low participation, we could repeat the teaching session to capture residents on rotations that made attendance a challenge (night shifts, intensive care units, distant hospitals or clinics, etc.). It should be noted that our postcourse survey asked specifically about abilities and comfort “after the teaching intervention,” which may have biased results favorably; however, it is reassuring that the 4-month results were similar to those directly after the course, with durable and continued benefit.

Using shoulder and knee models was a critical component of our teaching intervention. These models allowed the residents to place a simulation needle into an anatomically correct joint model with resistance similar to what would be experienced in live patients. The models we used were smaller than the average patient, and this may have affected the feel of the joint injection. However, we focused on using external and palpable landmarks to prepare the trainees for this difference and had the trainees draw the landmark and bony structures on each other. Additional models for each joint would have allowed the residents further practice but would have come at additional cost. Ideally, a practical exam later in the training year could be added to monitor procedural competency.

Our workshop-style teaching session for residents and supervising outpatient clinic faculty led to significant and durable increases in resident's confidence of their knowledge and ability to perform two common joint injections. Further development of this model may increase clinical performance and practice confidence and help make this relatively simple outpatient procedure more widely accessible to patients with musculoskeletal issues.

## Appendices

Teaching Flow Plan.docxJoint Injections.pptxJoint Injection Handout.docxPreworkshop Questionnaire.docxPostworkshop Questionnaire.docxFour-Month Follow-Up Questionnaire.docx
All appendices are peer reviewed as integral parts of the Original Publication.

## References

[1] Houston TK, Connors RL, Cutler N, Nidiry MA A primary care musculoskeletal clinic for residents: success and sustainability. J Gen Intern Med. 2004;19(5):524–529. 10.1111/j.1525-1497.2004.30173.x 15109317 PMC1492313

[2] Jolly M, Curran JJ Underuse of intra-articular and periarticular corticosteroid injections by primary care physicians: discomfort with the technique. J Clin Rheumatol. 2003;9(3):187–192. 10.1097/01.rhu.0000073587.90836.23 17041456

[3] Arroll B, Goodyear-Smith F Corticosteroid injections for osteoarthritis of the knee: meta-analysis. BMJ. 2004;328(7444):869 10.1136/bmj.38039.573970.7c 15039276 PMC387479

[4] Blair B, Rokito AS, Cuomo F, Jarolem K, Zuckerman JD Efficacy of injections of corticosteroids for subacromial impingement syndrome. J Bone Joint Surg Am. 1996;78(11):1685–1689. 10.2106/00004623-199611000-00007 8934482

[5] Bonds DE, Mychaleckyj JC, Watkins R, Palla S, Extrom P Ambulatory care skills: do residents feel prepared? Med Educ Online. 2002;7(1):4536 10.3402/meo.v7i.4536 28253755

[6] Wickstrom GC, Kelley DK, Keyserling TC, et al Confidence of academic general internists and family physicians to teach ambulatory procedures. J Gen Intern Med. 2000;15(6):353–360. 10.1046/j.1525-1497.2000.04109.x 10886468 PMC1495470

[7] Barilla-LaBarca ML, Tsang JC, Goldsmith M, Furie R Design, implementation, and outcome of a hands-on arthorocentesis workshop. J Clin Rhuematol. 2009;15(6):275–279. 10.1097/rhu.0b013e3181b68a62 19734731

[8] Vogelgesang SA, Karplus TM, Kreiter CD An instructional program to facilitate teaching joint/soft-tissue injection and aspiration. J Gen Intern Med. 2002;17(6):441–445. 10.1046/j.1525-1497.2002.10310.x 12133158 PMC1495064

[9] Wilcox T, Oyler J, Harada C, Utset T Musculoskeletal exam and joint injection training for internal medicine residents. J Gen Intern Med. 2006;21(5):521–523. 10.1111/j.1525-1497.2006.00442.x 16704403 PMC1484790

[10] Gormley GJ, Steele WK, Stevenson M, et al A randomised study of two training programmes for general practitioners in the techniques of shoulder injection. Ann Rheum Dis. 2003;62(10):1006–1009. 10.1136/ard.62.10.1006 12972483 PMC1754313

[11] Denizard-Thompson N, Feiereisel KB, Pedley CF, Burns C, Campos C Musculoskeletal basics: the shoulder and the knee workshop for primary care residents. MedEdPORTAL. 2018;14:10749 10.15766/mep_2374-8265.10749 30800949 PMC6342436

[12] Janssen SK, VanderMeulen SP, Brown D Hands-on lightly embalmed cadaver lab for teaching knee aspiration/injection. MedEdPORTAL. 2012;8:9187 10.15766/mep_2374-8265.9187

[13] Seifert MK, Pitzer M Does a dedicated joint injection teaching session improve resident confidence in performing joint injections? Poster presented at: American Medical Society for Sports Medicine Annual Meeting; April 15, 2015; Hollywood, FL.

[14] Chiowchanwisawakit P, Ratanarat R, Srinonprasert V Improving sixth year medical students' performance in knee arthrocentesis using a synthetic knee model. Int J Rheum Dis. 2015;18(7):742–750. 10.1111/1756-185x.12664 25988953

[15] Kwon SY, Hong SH, Kim ES, Park HJ, You Y, Kim YH The efficacy of lumbosacral spine phantom to improve resident proficiency in performing ultrasound-guided spinal procedure. Pain Med. 2015;16(12):2284–2291. 10.1111/pme.12870 26234900

[16] Ford SE, Patt JC, Scannell BP A comprehensive, high-quality orthopedic intern surgical skills program. J Surg Educ. 2016;73(4):553–558. 10.1016/j.jsurg.2016.03.008 27142722

[17] Battistone MJ, Barker AM, Grotzke MP, et al Effectiveness of an interprofessional and multidisciplinary musculoskeletal training program. J Grad Med Educ. 2016;8(3):398–404. 10.4300/jgme-d-15-00391.1 27413444 PMC4936859

[18] Leopold SS, Morgan HD, Kadel NJ, Gardner GC, Schaad DC, Wolf FM Impact of educational intervention on confidence and competence in the performance of a simple surgical task. J Bone Joint Surg Am. 2005;87(5):1031–1037. 10.2106/jbjs.d.02434 15866966

[19] Lenchus J, Issenberg SB, Murphy D, et al A blended approach to invasive bedside procedural instruction. Med Teach. 2011;33(2):116–123. 10.3109/0142159x.2010.509412 20874027

[20] Berman JR, Ben-Artzi A, Fisher MC, Bass AR, Pillinger MH A comparison of arthrocentesis teaching tools: cadavers, synthetic joint models, and the relative utility of different educational modalities in improving trainees' comfort with procedures. J Clin Rheumatol. 2012;18(4):175–179. 10.1097/rhu.0b013e318258259e 22647857

