# Supplementary material for: Improving Internal Medicine Resident Comfort With Shoulder and Knee Joint Injections Using an Injection Workshop
Source: MedEdPORTAL. 2020 Sep 28;16:10979. doi: 10.15766/mep_2374-8265.10979 (PMC7521064; doi:10.15766/mep_2374-8265.10979)
Supplement: Supplementary file 1 — Teaching Flow Plan.docxJoint Injections.pptxJoint Injection Handout.docxPreworkshop Questionnaire.docxPostworkshop Questionnaire.docxFour-Month Follow-Up Questionnaire.docx [file mep_2374-8265.10979-s001.zip › A. Teaching Flow Plan.docx]

**Joint Injection Teaching Flow Plan**

25-30 residents

~2 minutes per resident with shoulder and with knee

2 knee and 2 shoulder models

Timeline:

1:00-1:10 Introduction, explanation of curriculum change, explanation of surveys

1:10-1:20 Residents fill out pre-participation survey

1:20-1:50 Powerpoint presentation

1:50-1:55 Break in to groups of 3-4 (should be 7-8 groups of residents), pass out handouts

| Time | Knee Models (2) | Shoulder Models (2) |
| --- | --- | --- |
| 1:55-2:10 | Groups 1 and 2 | Groups 3 and 4 |
| 2:10-2:25 | Groups 5 and 6 | Groups 7 and 8 |
| 2:25-2:40 | Groups 3 and 4 | Groups 1 and 2 |
| 2:40-2:55 | Groups 7 and 8 | Groups 5 and 6 |

Groups not involved with joint model will be drawing landmarks on each other’s shoulders and knee, asking for clarification from presenters

3:15-3:30 Residents fill out post-participation survey
