# Supplementary material for: Improving Internal Medicine Resident Comfort With Shoulder and Knee Joint Injections Using an Injection Workshop
Source: MedEdPORTAL. 2020 Sep 28;16:10979. doi: 10.15766/mep_2374-8265.10979 (PMC7521064; doi:10.15766/mep_2374-8265.10979)
Supplement: Supplementary file 1 — Teaching Flow Plan.docxJoint Injections.pptxJoint Injection Handout.docxPreworkshop Questionnaire.docxPostworkshop Questionnaire.docxFour-Month Follow-Up Questionnaire.docx [file mep_2374-8265.10979-s001.zip › B. Joint Injections.pptx]

## Slide 1
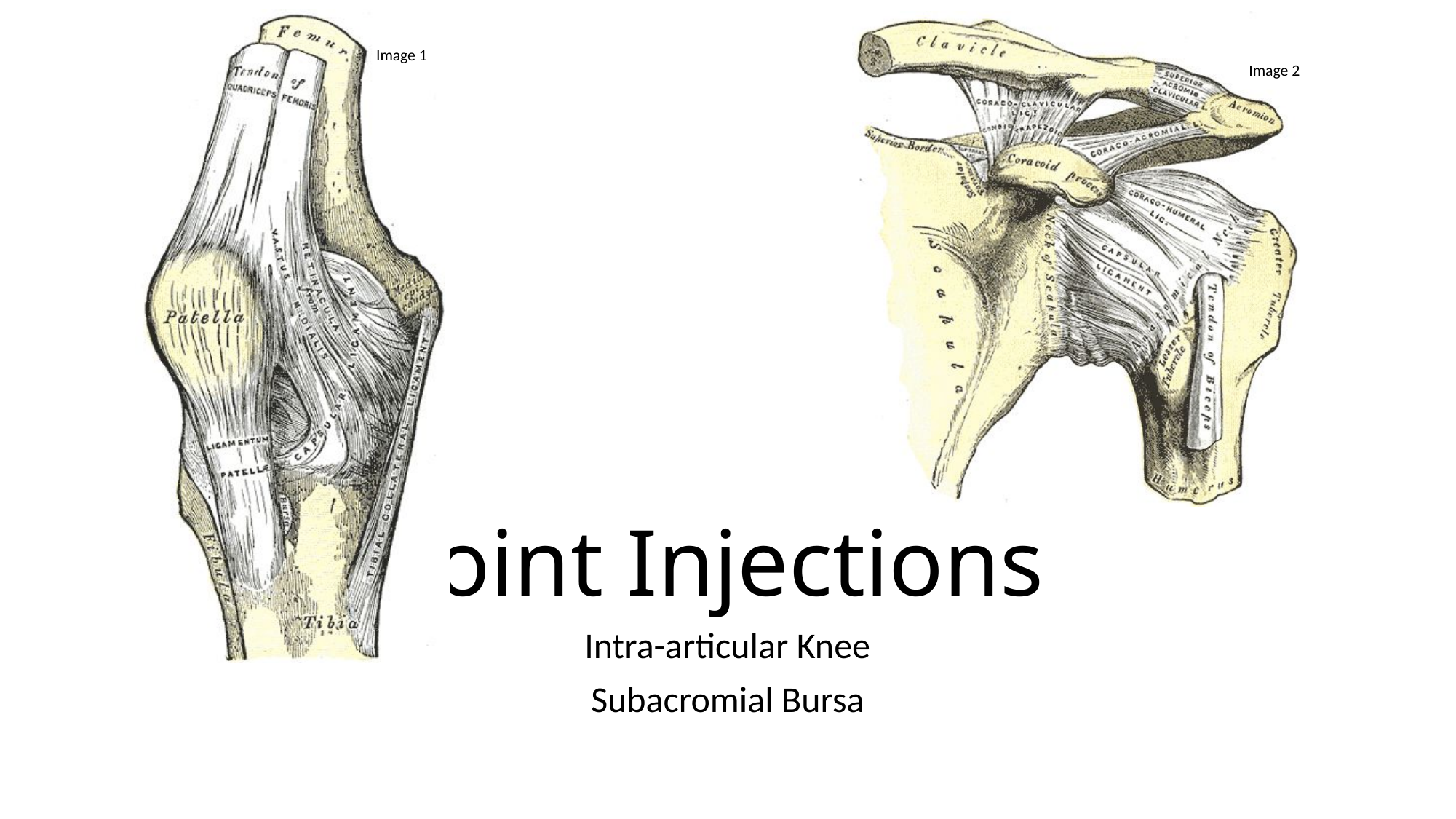

Image 1
Image 2
# Joint Injections
Intra-articular Knee
Subacromial Bursa

## Slide 2
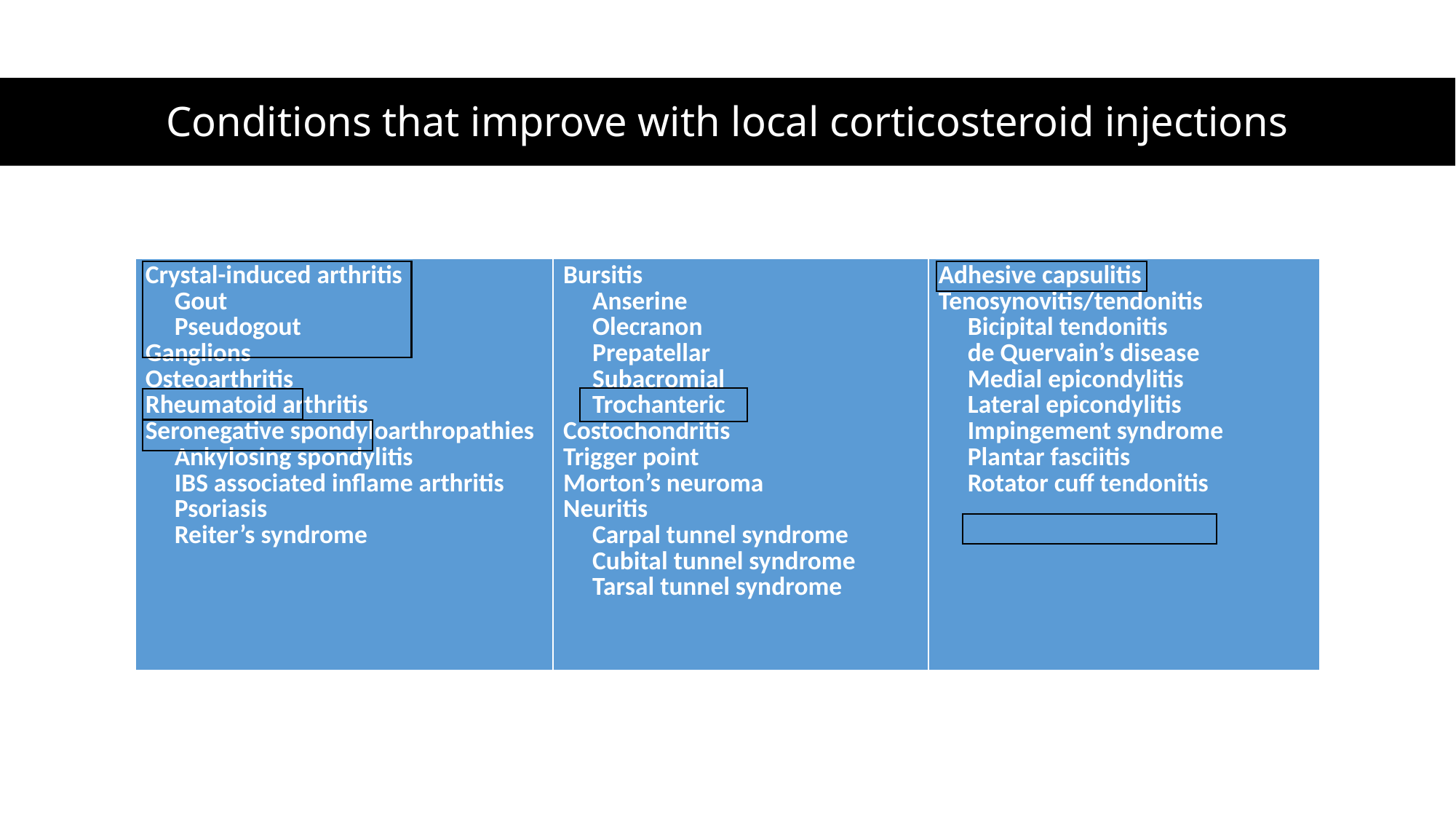

# Conditions that improve with local corticosteroid injections
| Crystal-induced arthritis Gout Pseudogout Ganglions Osteoarthritis Rheumatoid arthritis Seronegative spondyloarthropathies Ankylosing spondylitis IBS associated inflame arthritis Psoriasis Reiter’s syndrome | Bursitis Anserine Olecranon Prepatellar Subacromial Trochanteric Costochondritis Trigger point Morton’s neuroma Neuritis Carpal tunnel syndrome Cubital tunnel syndrome Tarsal tunnel syndrome | Adhesive capsulitis Tenosynovitis/tendonitis Bicipital tendonitis de Quervain’s disease Medial epicondylitis Lateral epicondylitis Impingement syndrome Plantar fasciitis Rotator cuff tendonitis |
| --- | --- | --- |

## Slide 3
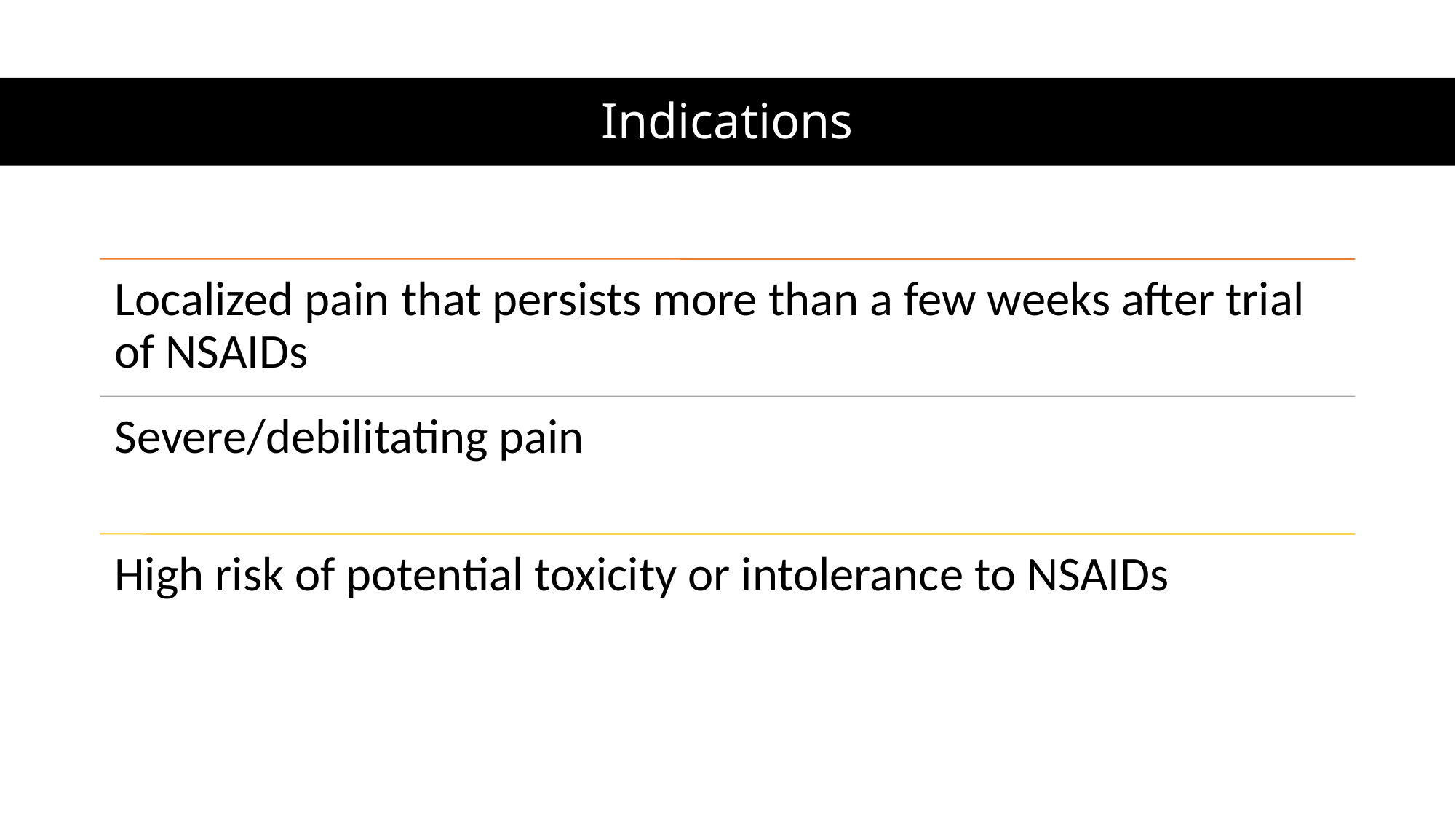

# Indications

## Slide 4
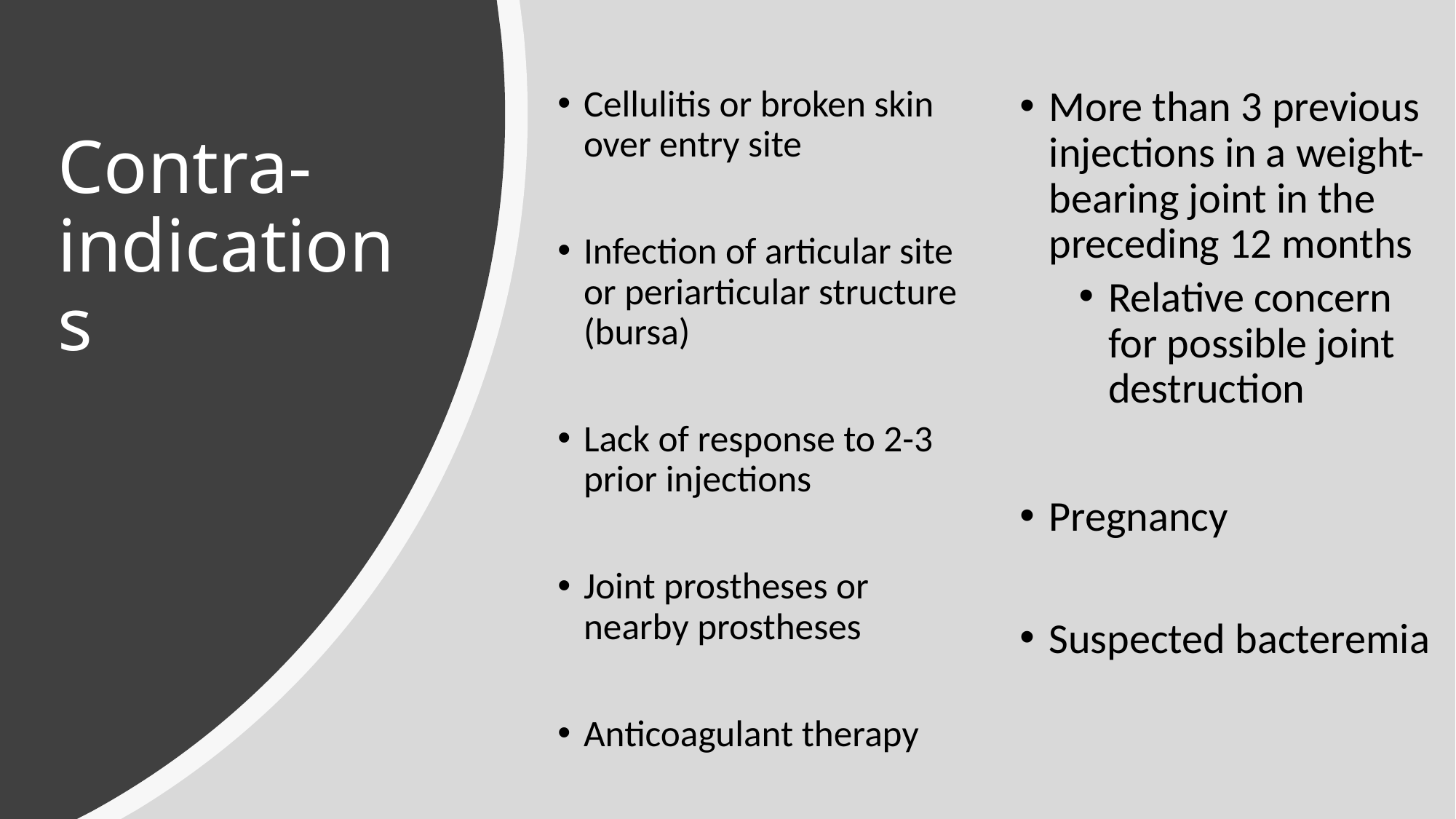

Cellulitis or broken skin over entry site
Infection of articular site or periarticular structure (bursa)
Lack of response to 2-3 prior injections
Joint prostheses or nearby prostheses
Anticoagulant therapy
More than 3 previous injections in a weight-bearing joint in the preceding 12 months
Relative concern for possible joint destruction
Pregnancy
Suspected bacteremia
# Contra-indications

## Slide 5
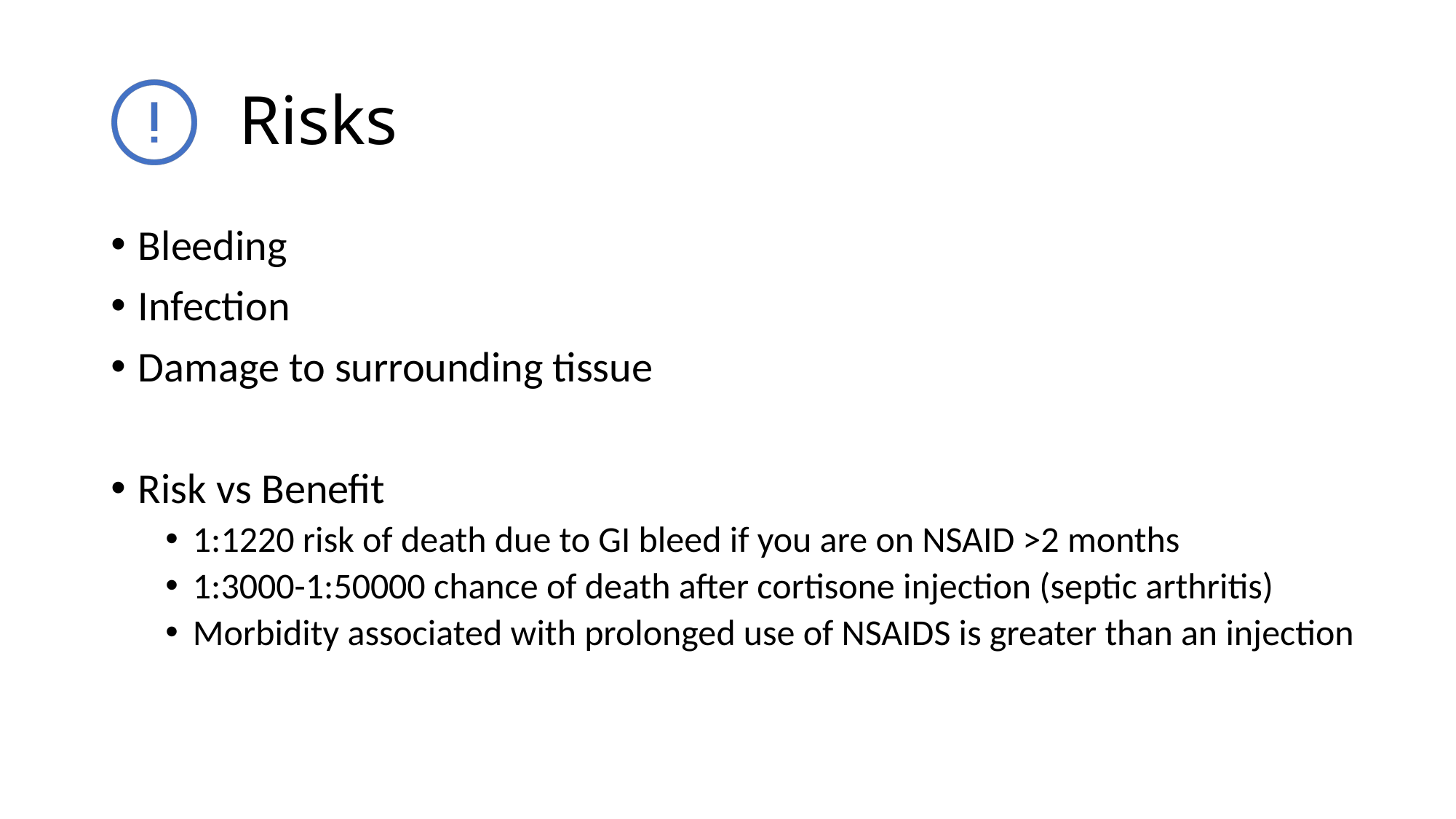

# Risks
Bleeding
Infection
Damage to surrounding tissue
Risk vs Benefit
1:1220 risk of death due to GI bleed if you are on NSAID >2 months
1:3000-1:50000 chance of death after cortisone injection (septic arthritis)
Morbidity associated with prolonged use of NSAIDS is greater than an injection

## Slide 6
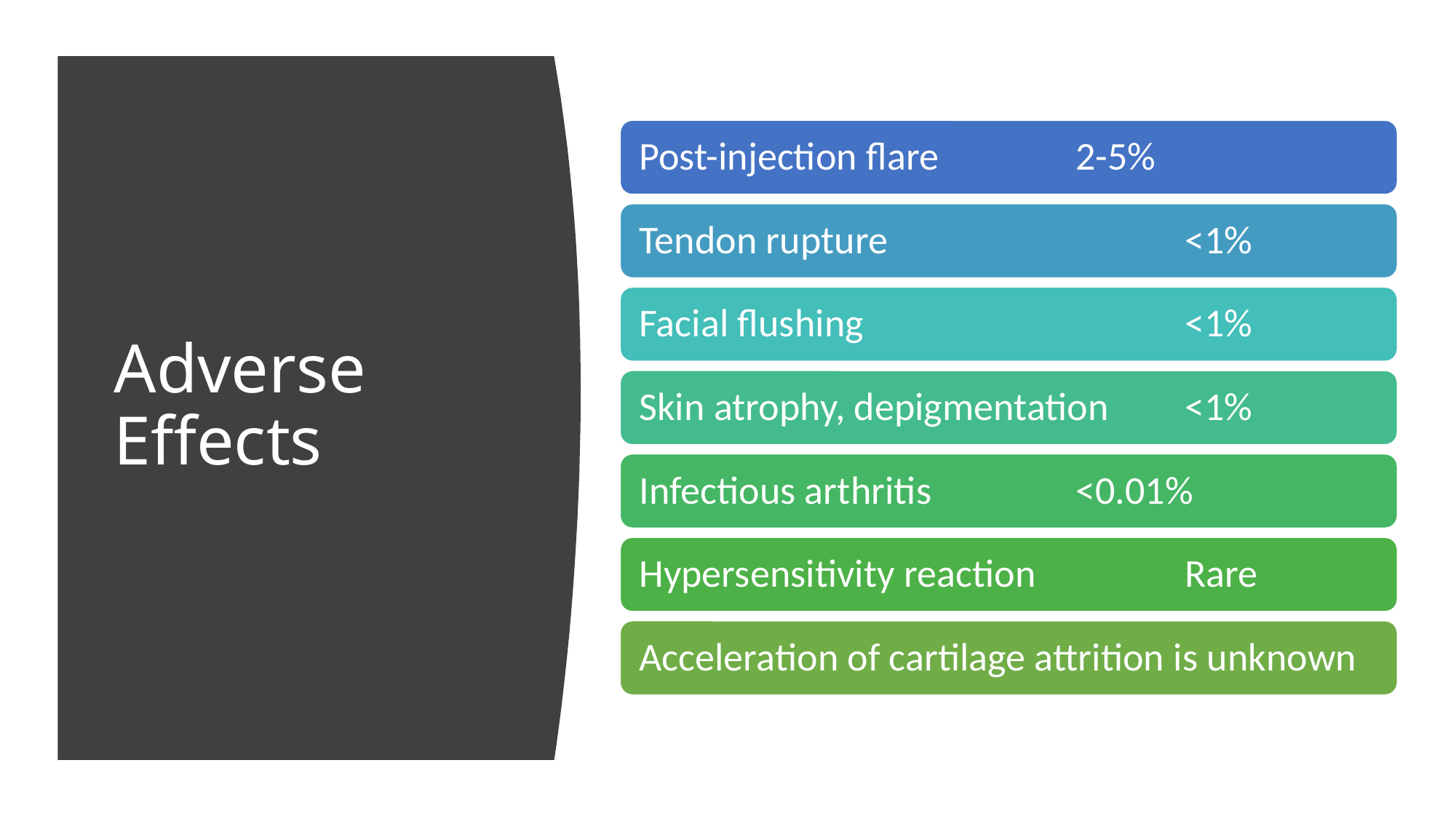

# Adverse Effects

## Slide 7
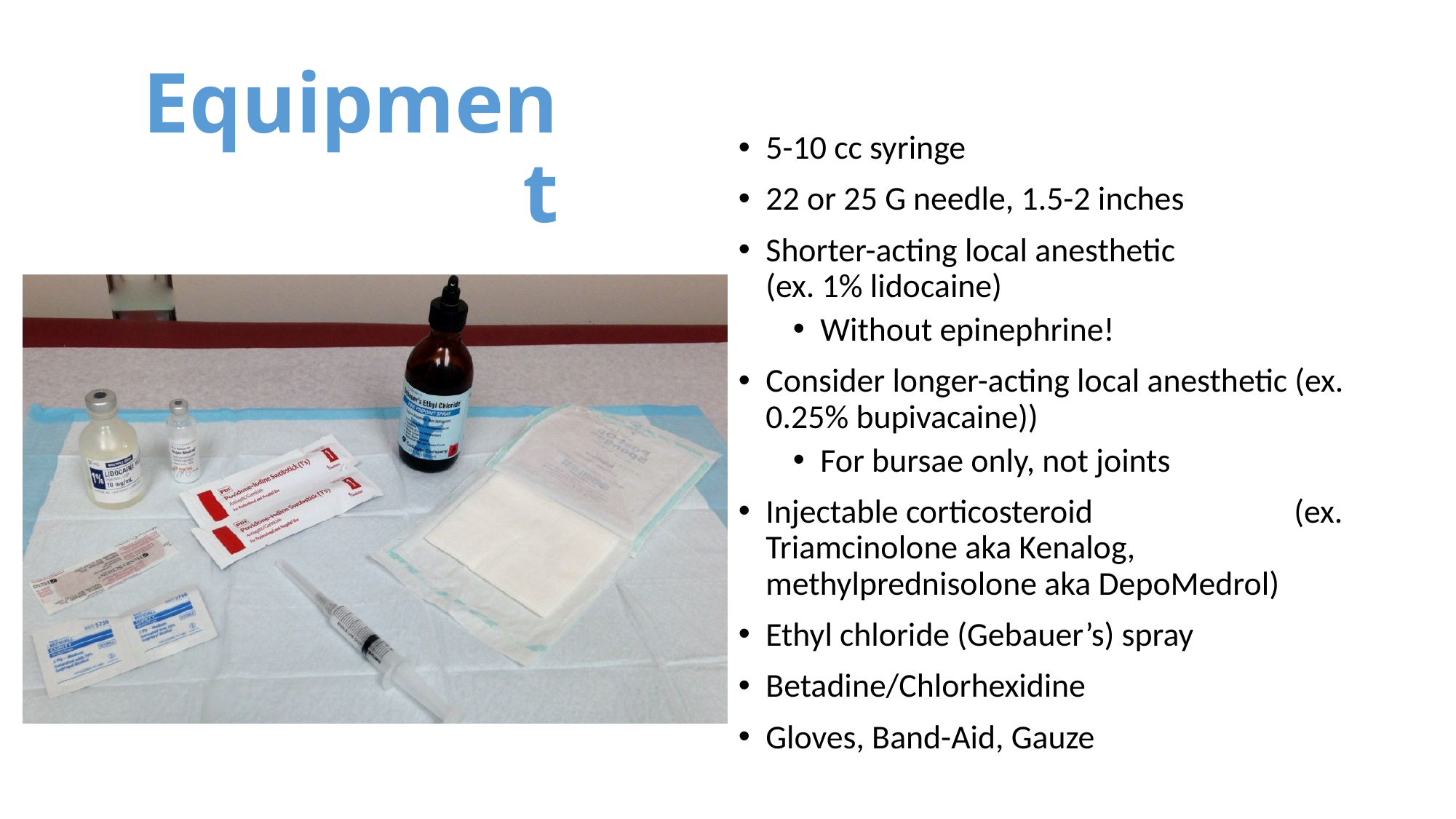

# Equipment
5-10 cc syringe
22 or 25 G needle, 1.5-2 inches
Shorter-acting local anesthetic (ex. 1% lidocaine)
Without epinephrine!
Consider longer-acting local anesthetic (ex. 0.25% bupivacaine))
For bursae only, not joints
Injectable corticosteroid (ex. Triamcinolone aka Kenalog, methylprednisolone aka DepoMedrol)
Ethyl chloride (Gebauer’s) spray
Betadine/Chlorhexidine
Gloves, Band-Aid, Gauze

## Slide 8
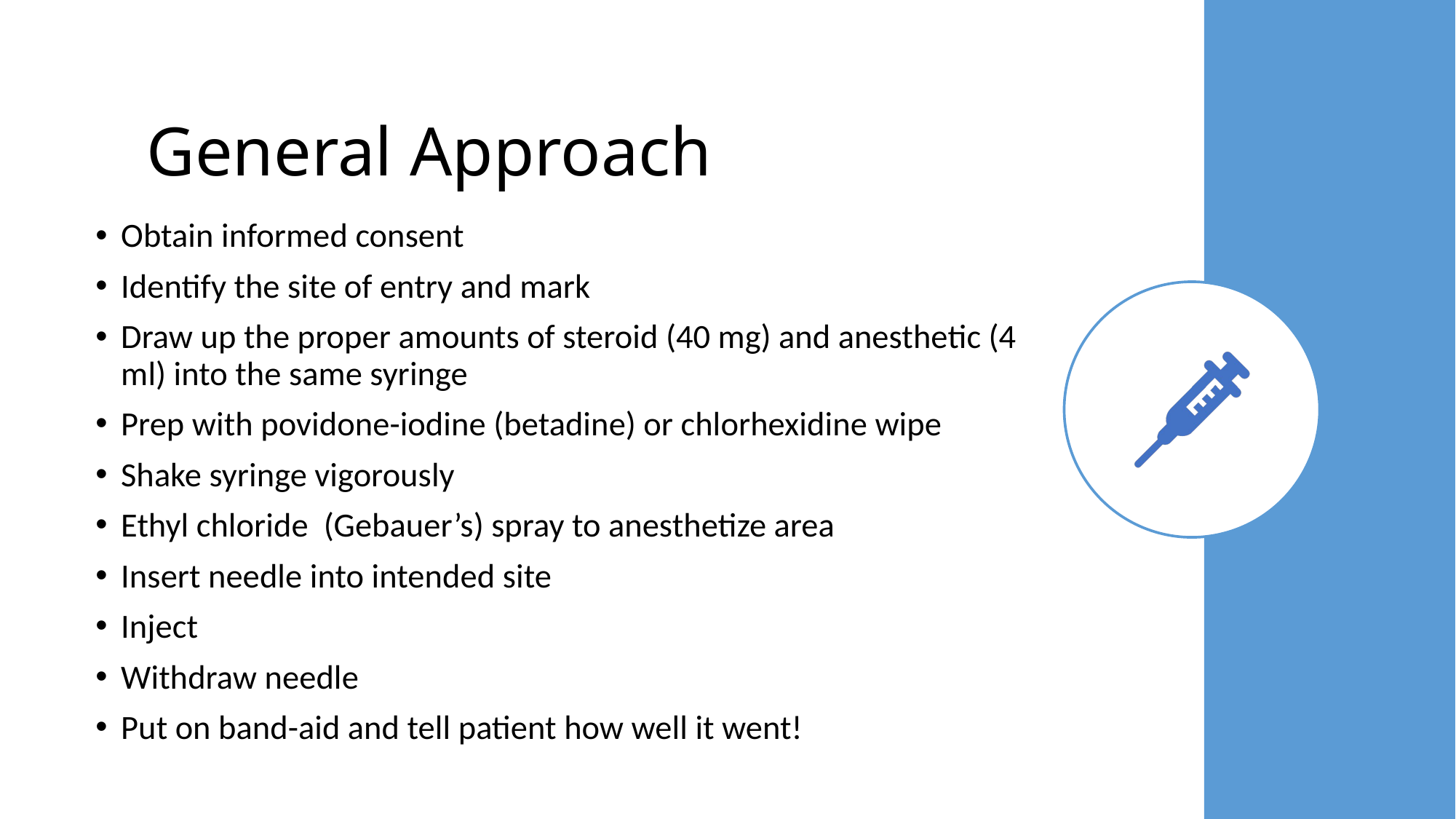

# General Approach
Obtain informed consent
Identify the site of entry and mark
Draw up the proper amounts of steroid (40 mg) and anesthetic (4 ml) into the same syringe
Prep with povidone-iodine (betadine) or chlorhexidine wipe
Shake syringe vigorously
Ethyl chloride (Gebauer’s) spray to anesthetize area
Insert needle into intended site
Inject
Withdraw needle
Put on band-aid and tell patient how well it went!

## Slide 9
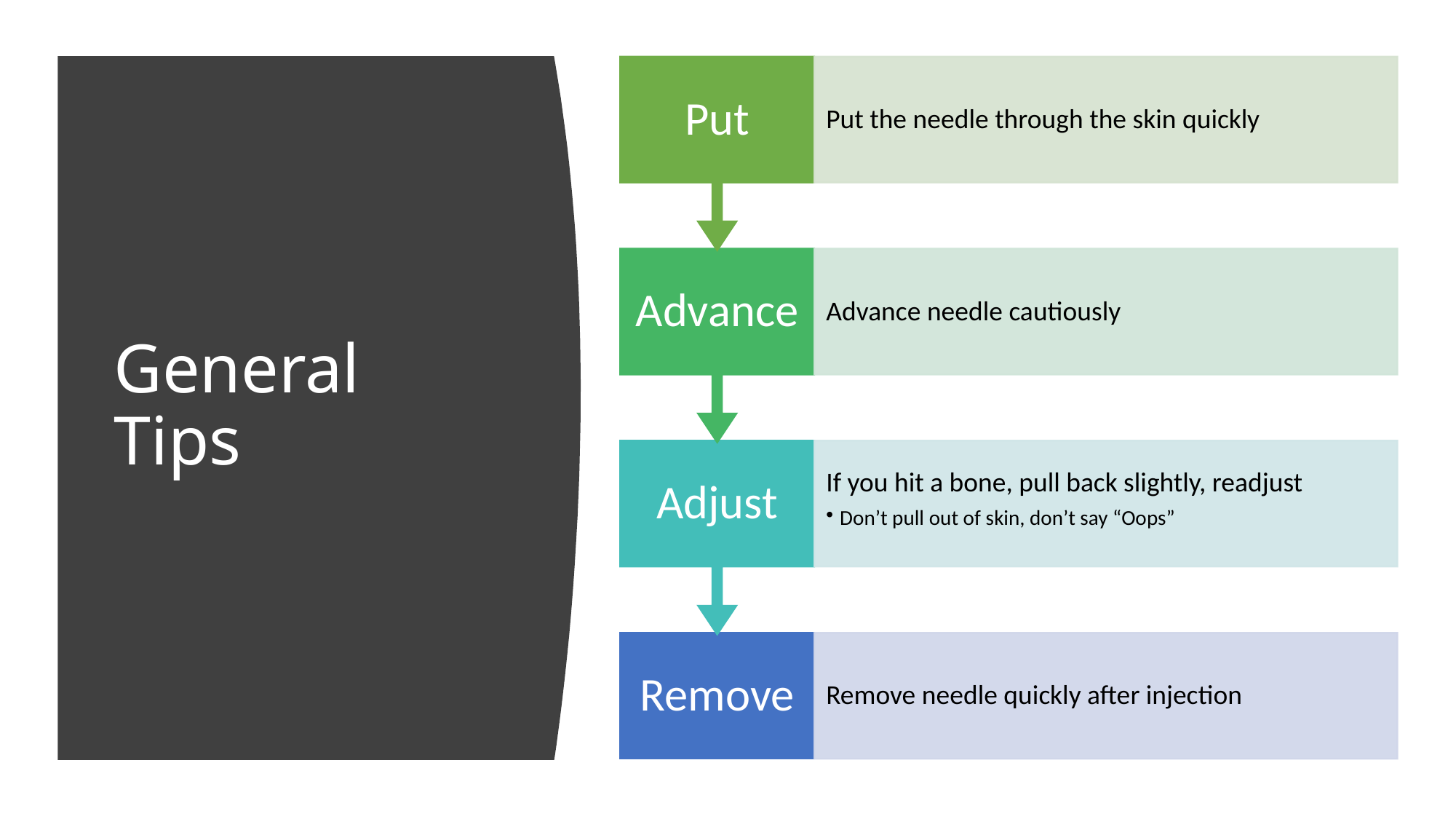

# General Tips

## Slide 10
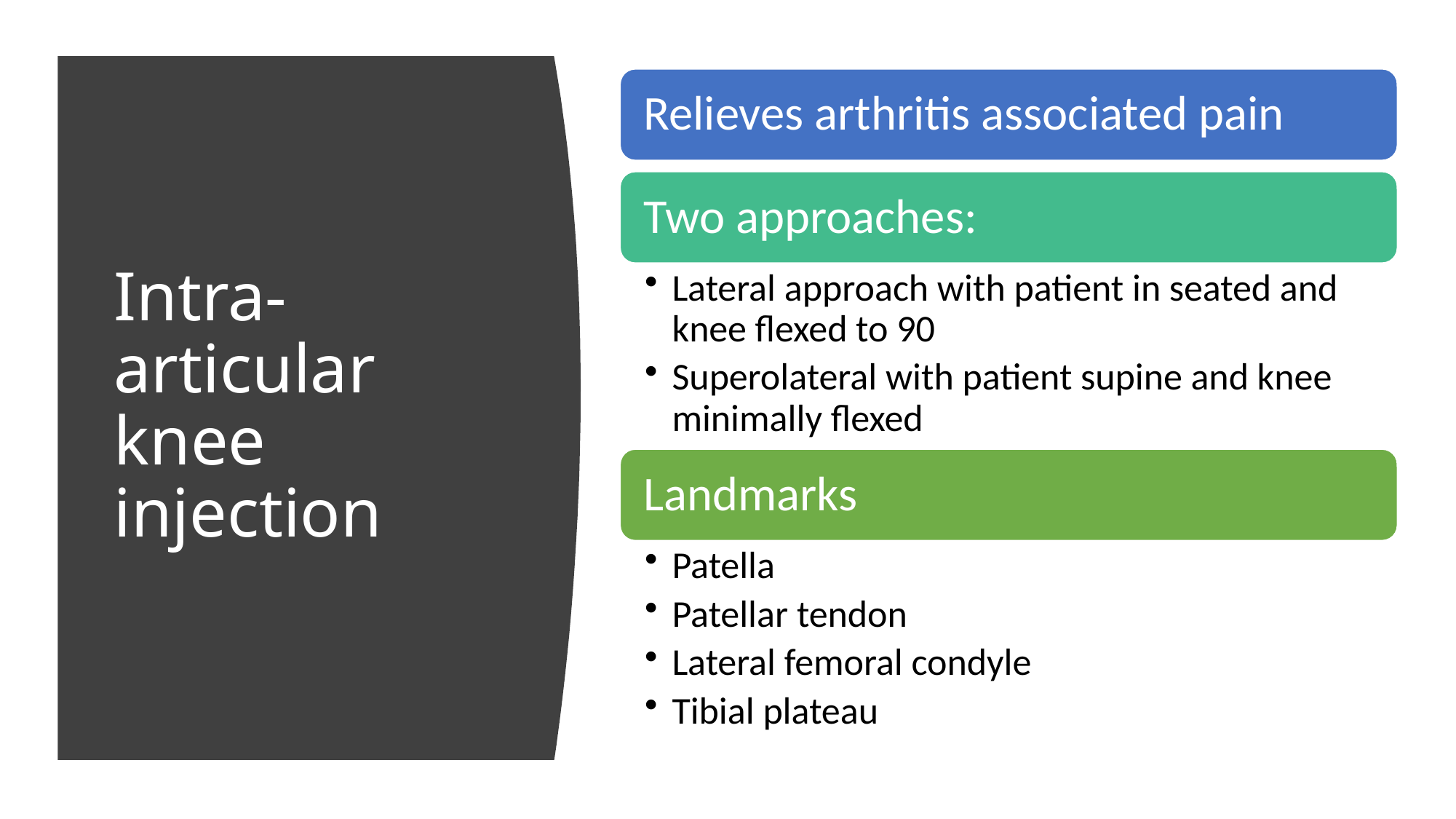

# Intra-articular knee injection

## Slide 11
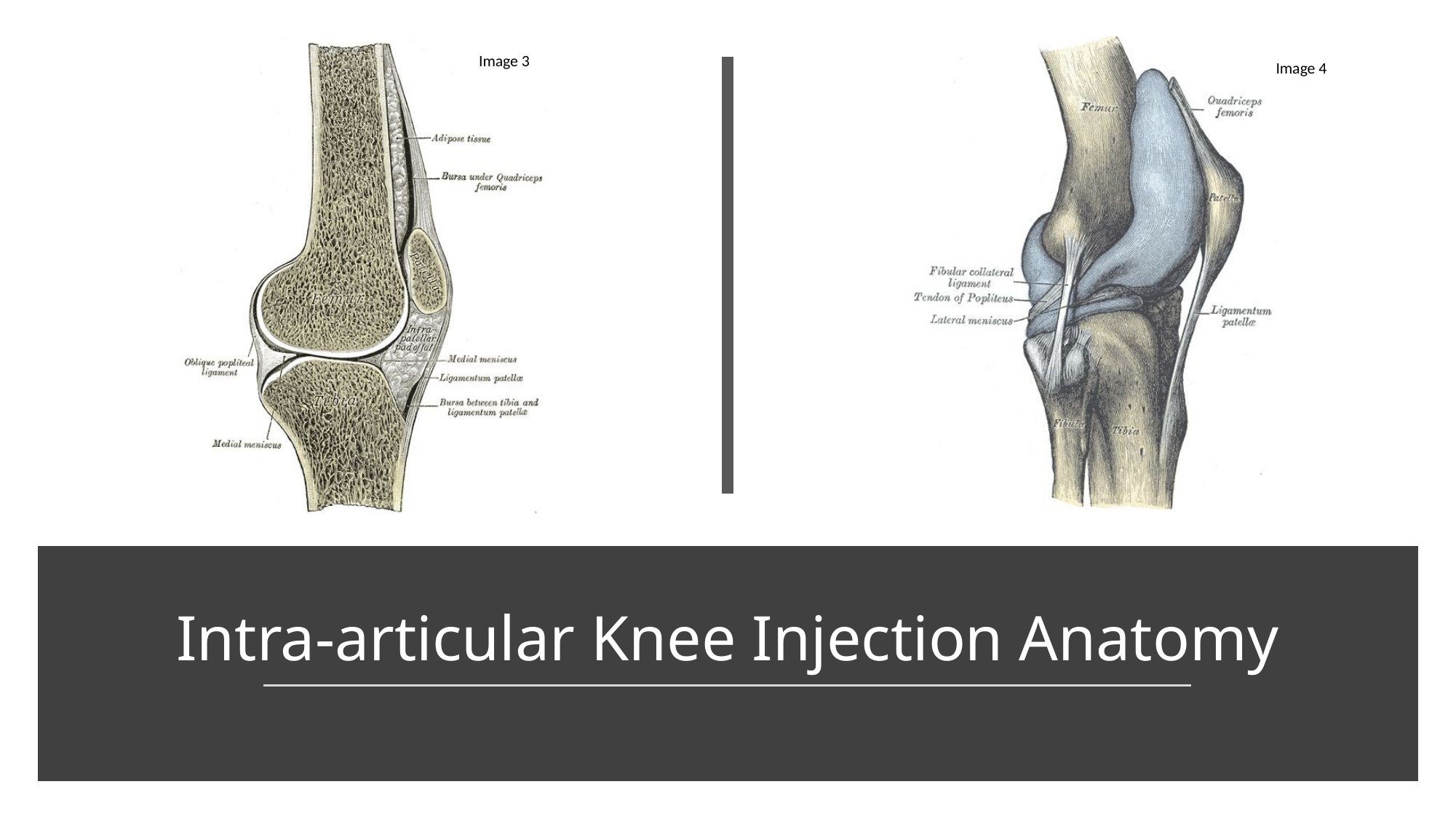

Image 3
Image 4
Image 5
# Intra-articular Knee Injection Anatomy

## Slide 12
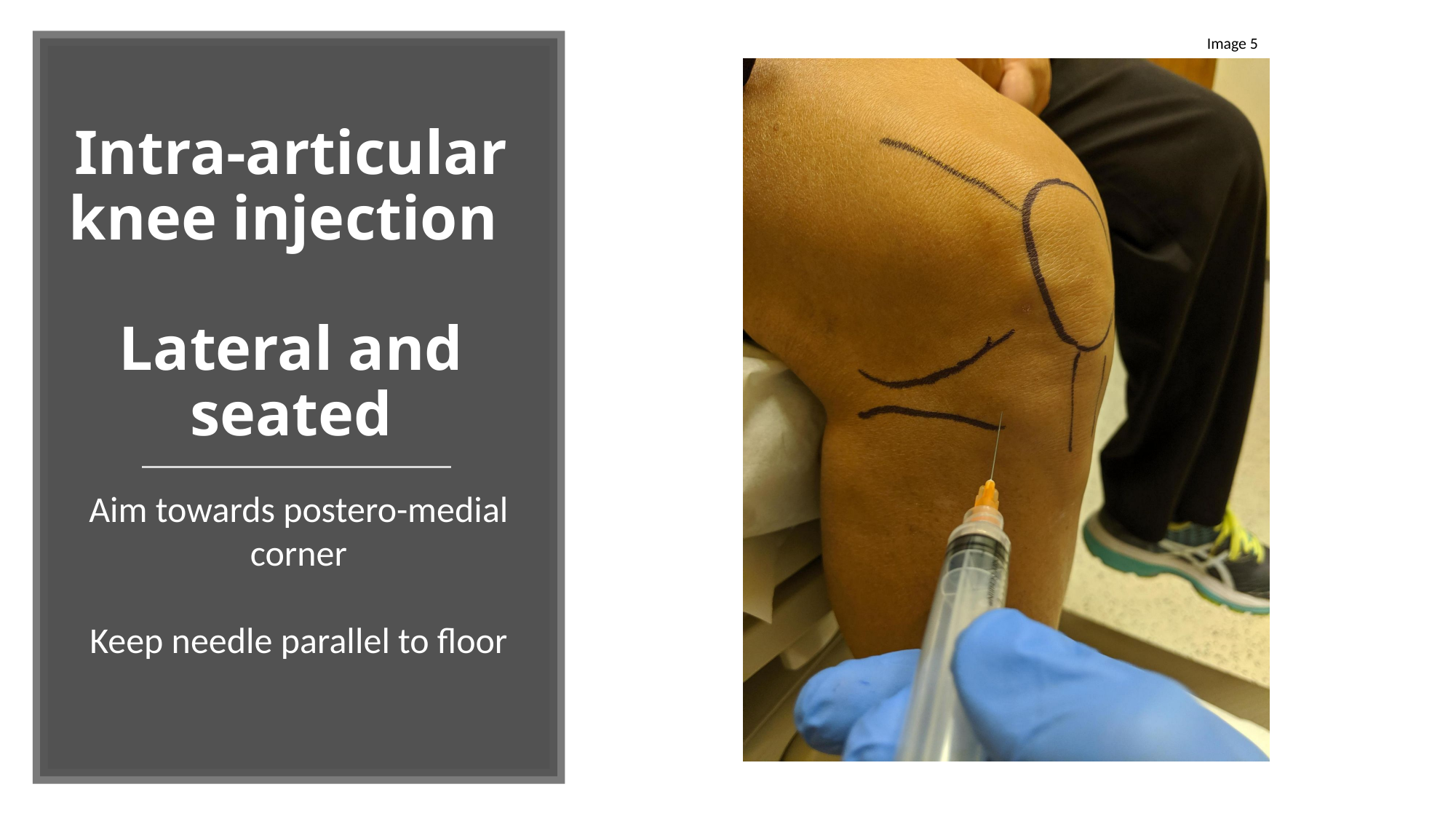

Image 5
# Intra-articular knee injection Lateral and seated
Aim towards postero-medial corner
Keep needle parallel to floor

## Slide 13
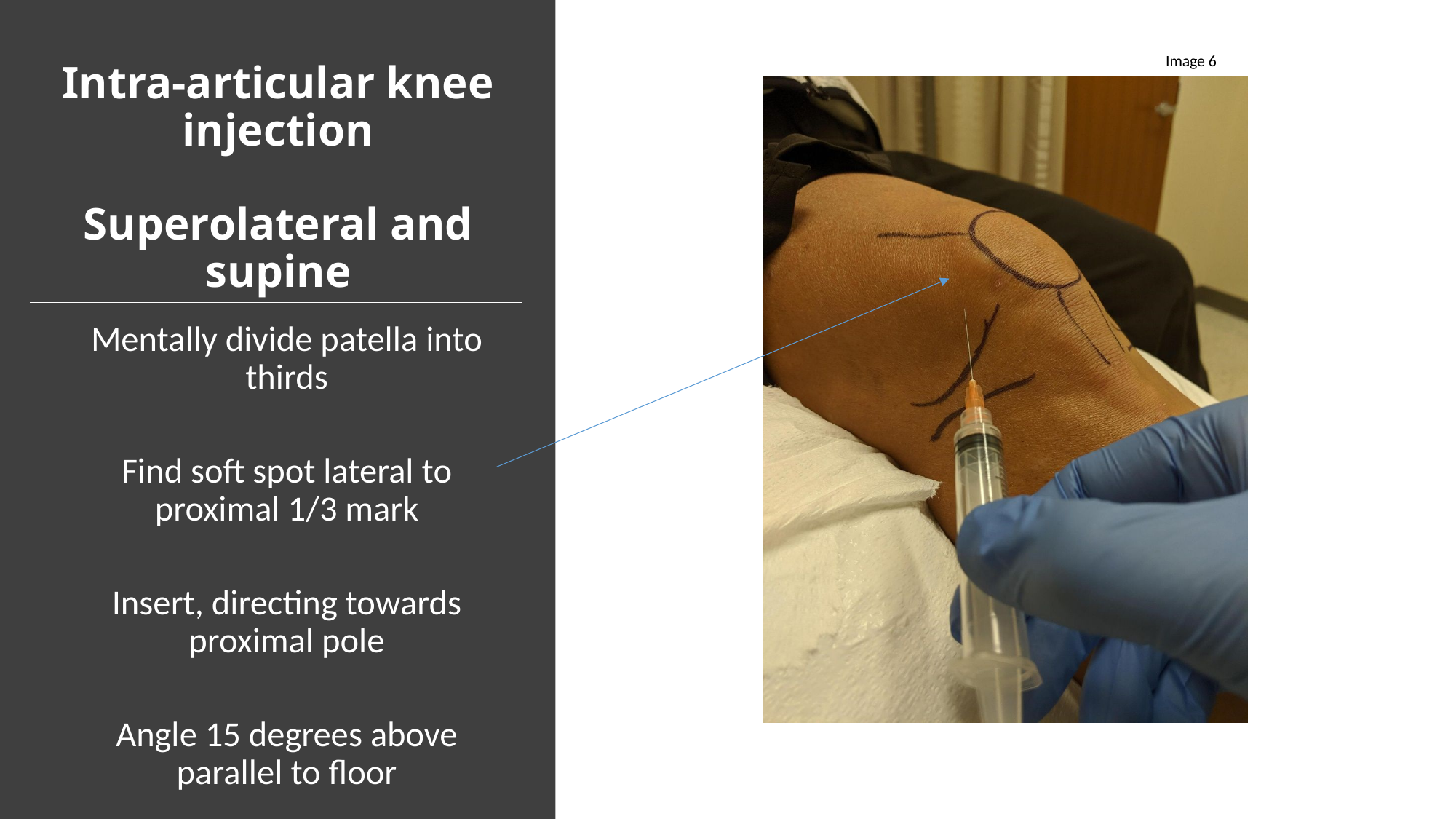

Image 6
# Intra-articular knee injection Superolateral and supine
Mentally divide patella into thirds
Find soft spot lateral to proximal 1/3 mark
Insert, directing towards proximal pole
Angle 15 degrees above parallel to floor

## Slide 14
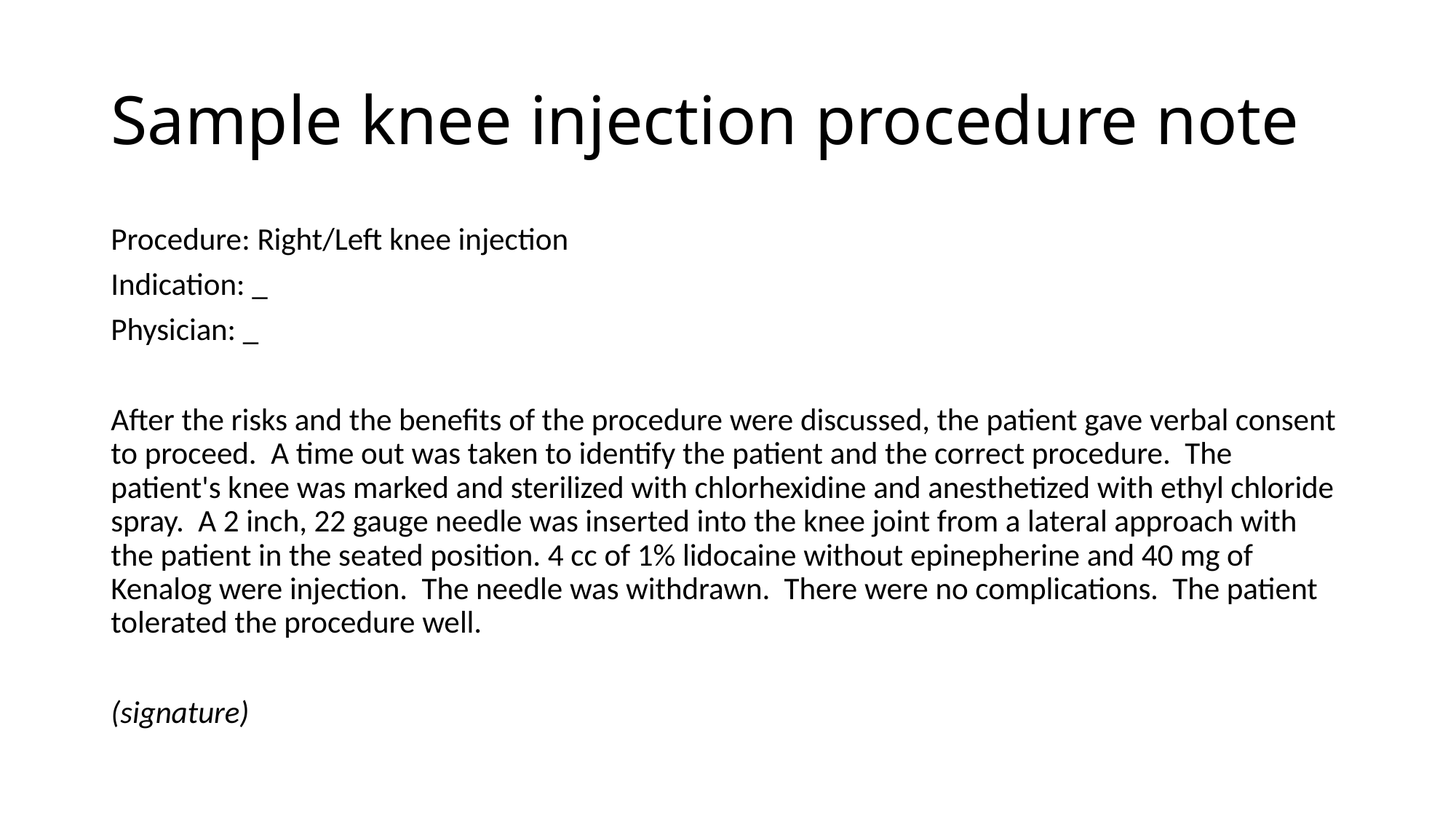

# Sample knee injection procedure note
Procedure: Right/Left knee injection
Indication: _
Physician: _
After the risks and the benefits of the procedure were discussed, the patient gave verbal consent to proceed. A time out was taken to identify the patient and the correct procedure. The patient's knee was marked and sterilized with chlorhexidine and anesthetized with ethyl chloride spray. A 2 inch, 22 gauge needle was inserted into the knee joint from a lateral approach with the patient in the seated position. 4 cc of 1% lidocaine without epinepherine and 40 mg of Kenalog were injection. The needle was withdrawn. There were no complications. The patient tolerated the procedure well.
(signature)

## Slide 15
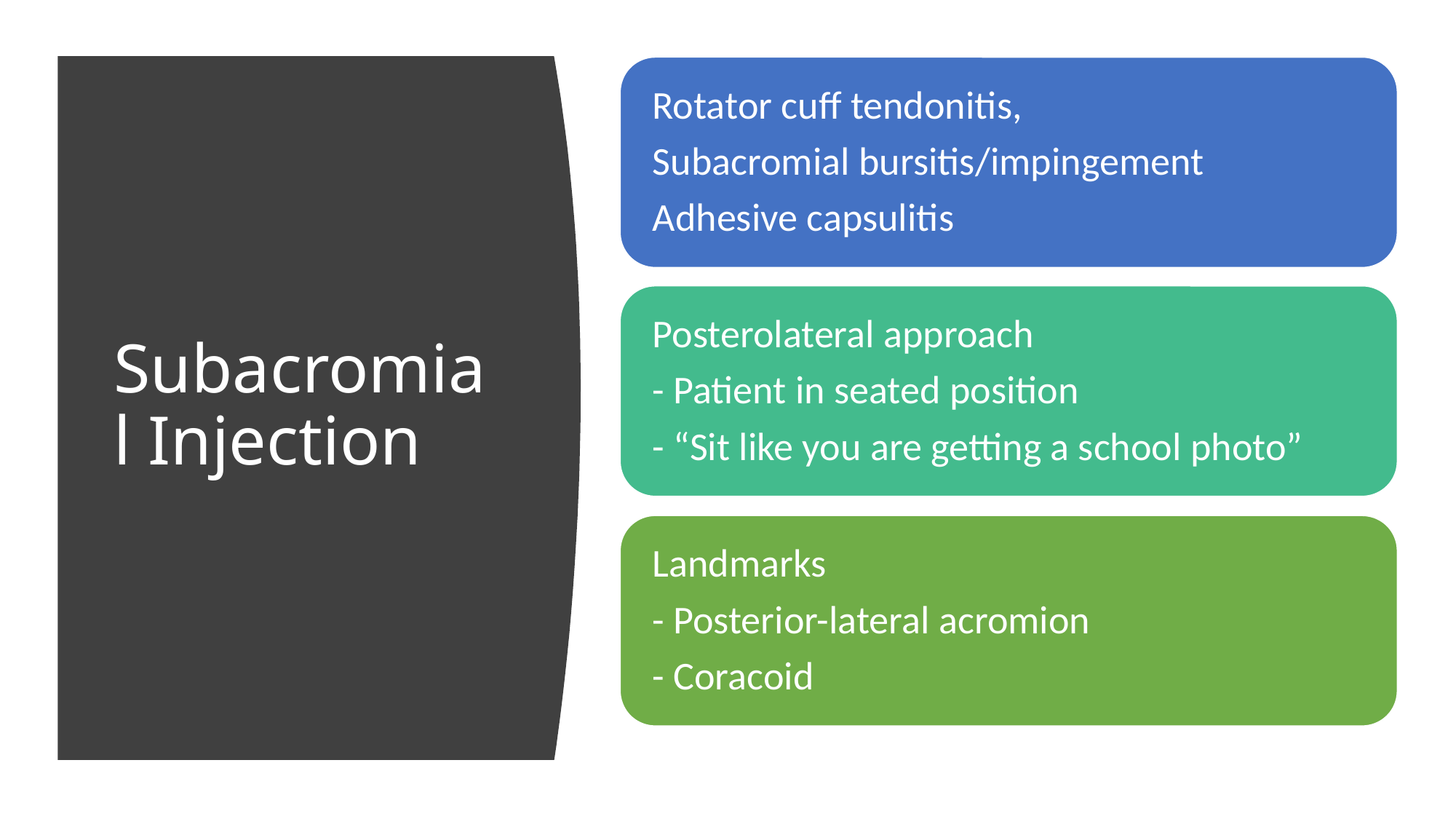

# Subacromial Injection

## Slide 16
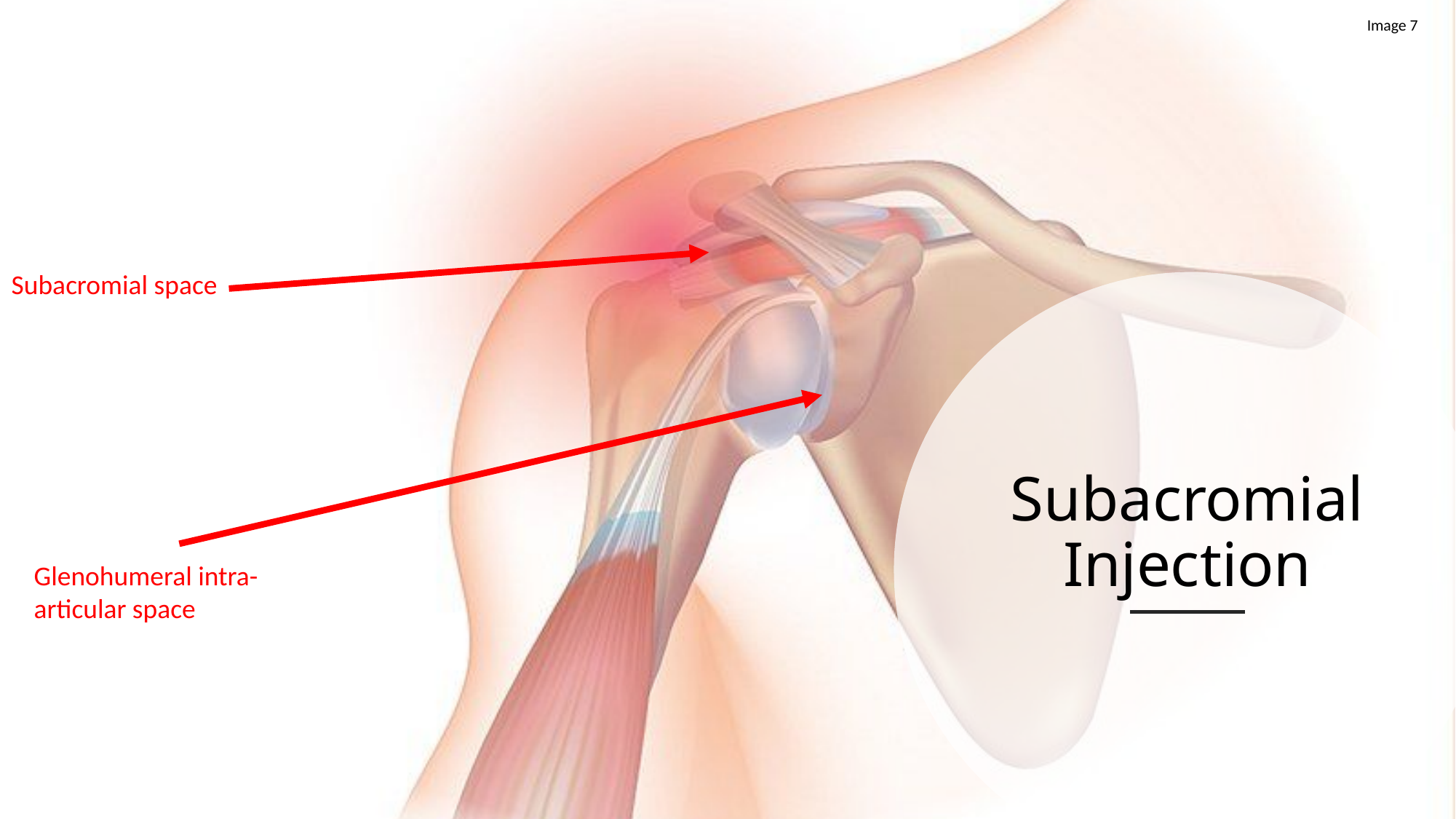

Image 7
Subacromial space
# Subacromial Injection
Glenohumeral intra-articular space

## Slide 17
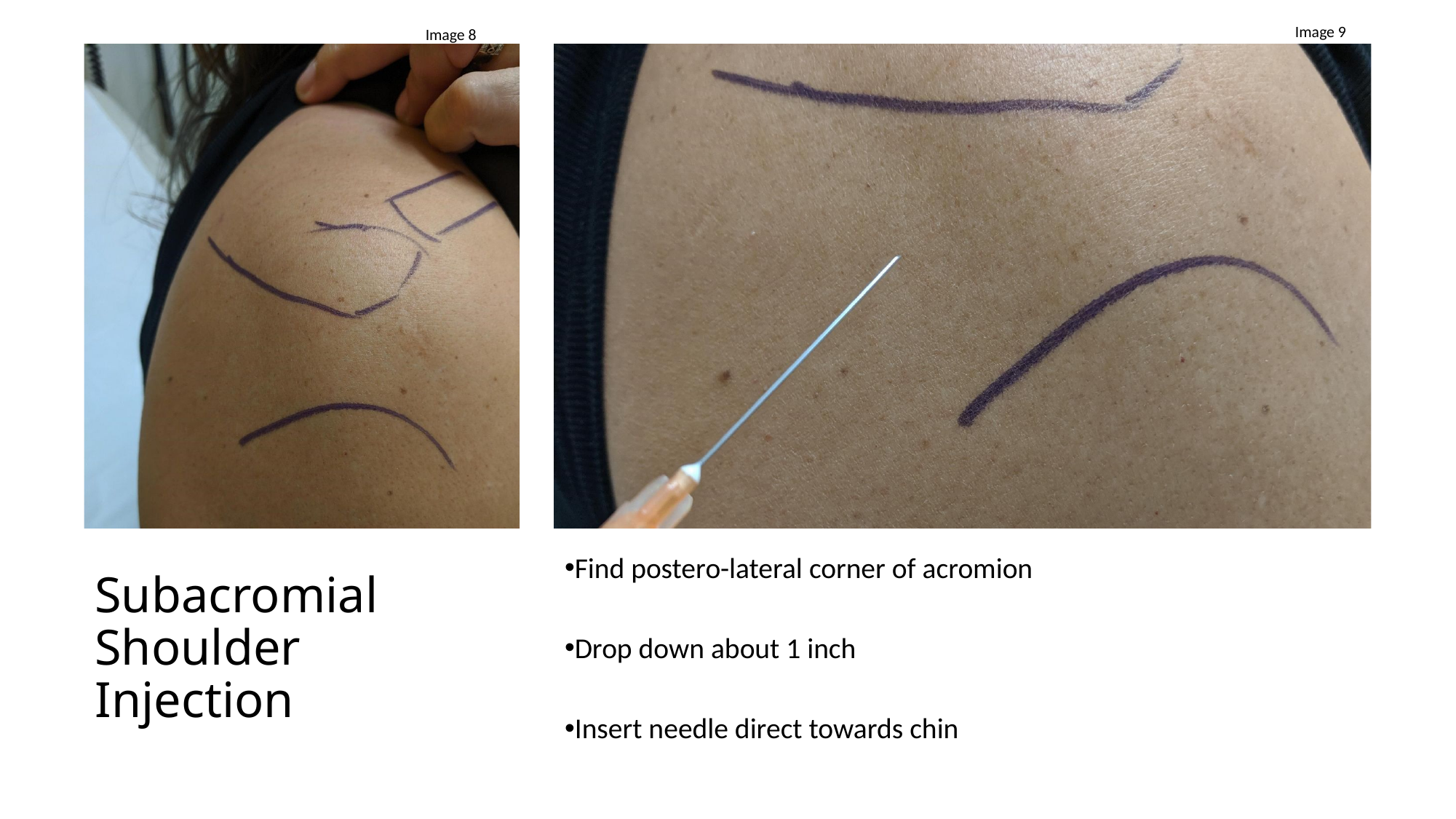

Image 9
Image 8
# Subacromial Shoulder Injection
Find postero-lateral corner of acromion
Drop down about 1 inch
Insert needle direct towards chin

## Slide 18
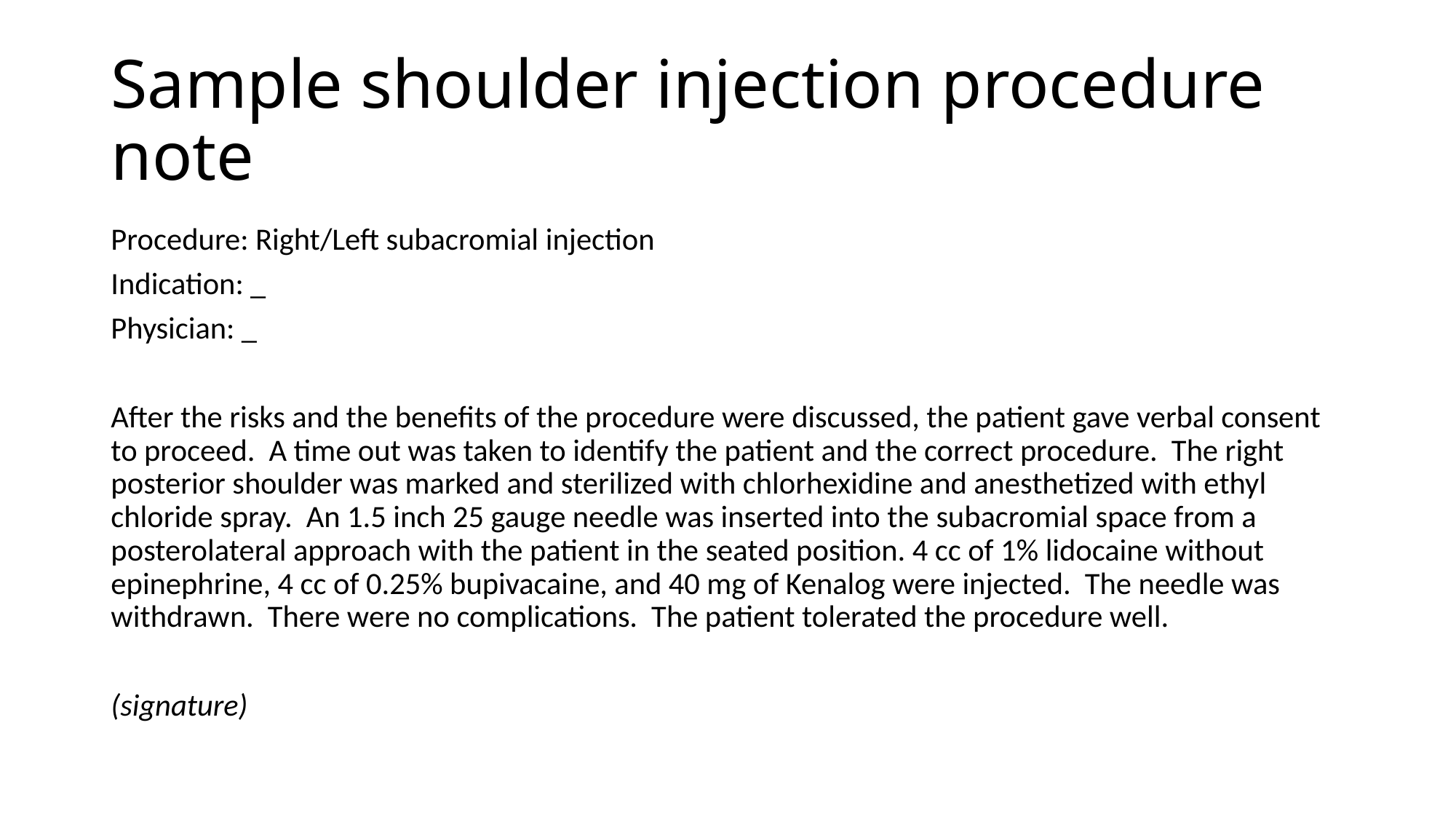

# Sample shoulder injection procedure note
Procedure: Right/Left subacromial injection
Indication: _
Physician: _
After the risks and the benefits of the procedure were discussed, the patient gave verbal consent to proceed. A time out was taken to identify the patient and the correct procedure. The right posterior shoulder was marked and sterilized with chlorhexidine and anesthetized with ethyl chloride spray. An 1.5 inch 25 gauge needle was inserted into the subacromial space from a posterolateral approach with the patient in the seated position. 4 cc of 1% lidocaine without epinephrine, 4 cc of 0.25% bupivacaine, and 40 mg of Kenalog were injected. The needle was withdrawn. There were no complications. The patient tolerated the procedure well.
(signature)

## Slide 19
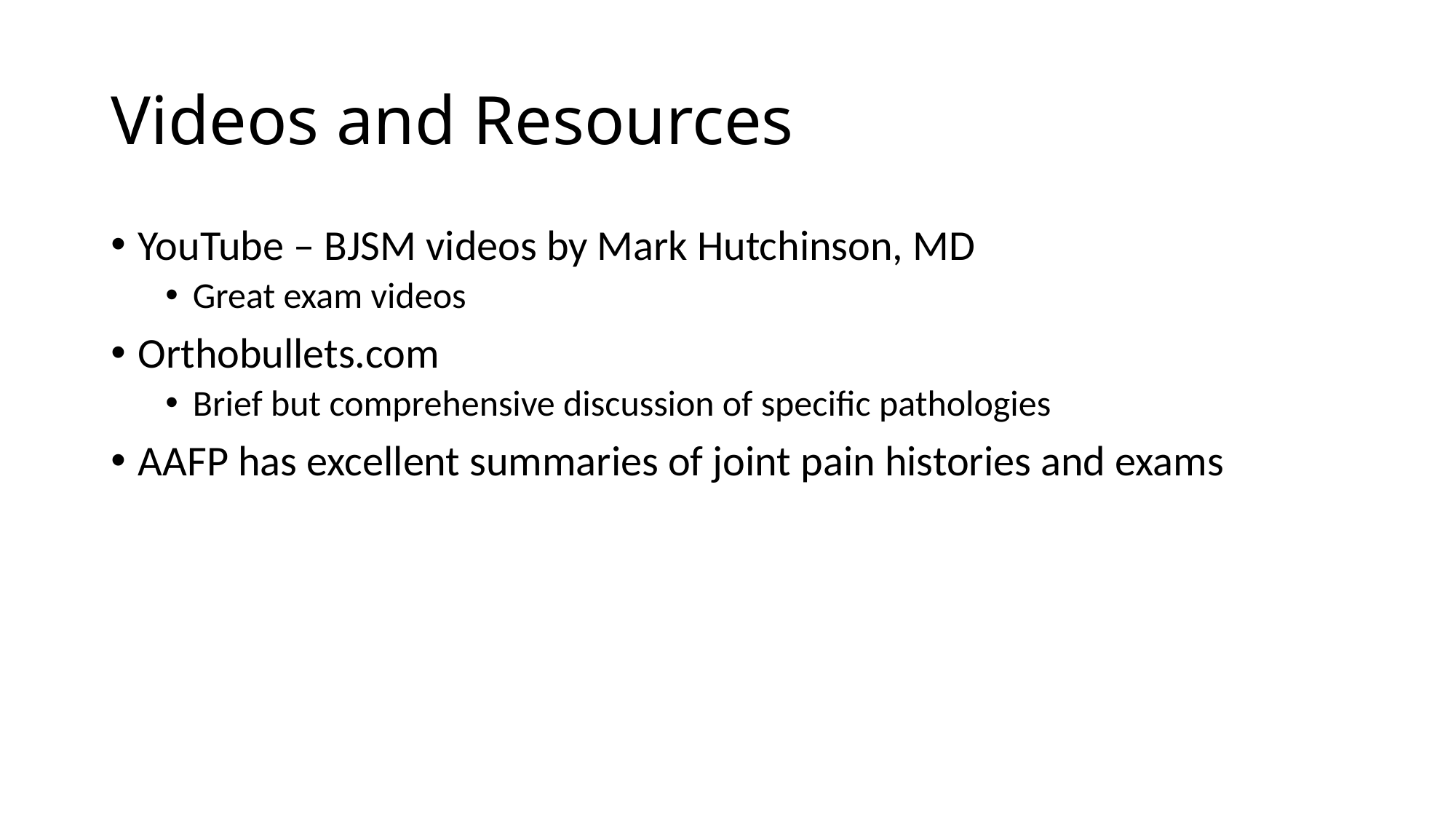

# Videos and Resources
YouTube – BJSM videos by Mark Hutchinson, MD
Great exam videos
Orthobullets.com
Brief but comprehensive discussion of specific pathologies
AAFP has excellent summaries of joint pain histories and exams

## Slide 20
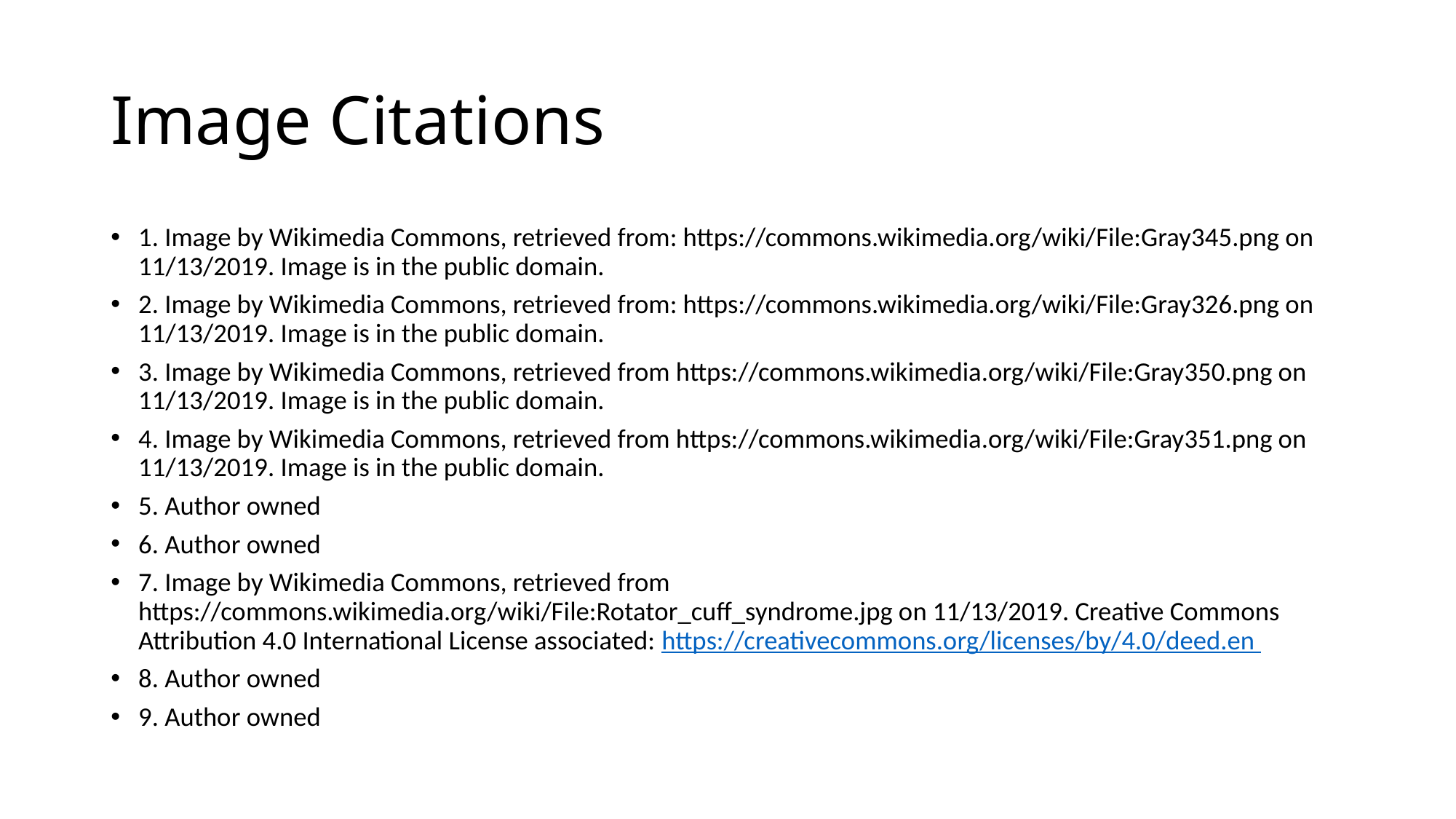

# Image Citations
1. Image by Wikimedia Commons, retrieved from: https://commons.wikimedia.org/wiki/File:Gray345.png on 11/13/2019. Image is in the public domain.
2. Image by Wikimedia Commons, retrieved from: https://commons.wikimedia.org/wiki/File:Gray326.png on 11/13/2019. Image is in the public domain.
3. Image by Wikimedia Commons, retrieved from https://commons.wikimedia.org/wiki/File:Gray350.png on 11/13/2019. Image is in the public domain.
4. Image by Wikimedia Commons, retrieved from https://commons.wikimedia.org/wiki/File:Gray351.png on 11/13/2019. Image is in the public domain.
5. Author owned
6. Author owned
7. Image by Wikimedia Commons, retrieved from https://commons.wikimedia.org/wiki/File:Rotator_cuff_syndrome.jpg on 11/13/2019. Creative Commons Attribution 4.0 International License associated: https://creativecommons.org/licenses/by/4.0/deed.en
8. Author owned
9. Author owned
