# Supplementary material for: Improving Internal Medicine Resident Comfort With Shoulder and Knee Joint Injections Using an Injection Workshop
Source: MedEdPORTAL. 2020 Sep 28;16:10979. doi: 10.15766/mep_2374-8265.10979 (PMC7521064; doi:10.15766/mep_2374-8265.10979)
Supplement: Supplementary file 1 — Teaching Flow Plan.docxJoint Injections.pptxJoint Injection Handout.docxPreworkshop Questionnaire.docxPostworkshop Questionnaire.docxFour-Month Follow-Up Questionnaire.docx [file mep_2374-8265.10979-s001.zip › C. Joint Injection Handout.docx]

**Internal Medicine – Sports Medicine Joint Injection Handout**

Indications for steroid joint or bursa injections

Localized pain > few weeks after trial of NSAIDs

Severe/debilitating pain

High risk of potential toxicity or intolerance to NSAIDs

Contra-indications for steroid joint or bursa injections (* indicates relative CI)

Cellulitis or broken skin over entry site Suspected bacteremia

Infection of articular site or periarticular structure (bursa) Prior injections haven’t worked

Joint prostheses or nearby prostheses Pregnancy*

> 3 previous injections in the preceding 12 months Anticoagulant therapy*

Relative concern for theoretic joint destruction

Risks and Benefits of steroid joint injections

| **Risks** | **Benefits** |
| --- | --- |
| Infection | Relief of pain |
| Bleeding | Decreased swelling |
| Damage to nearby tissue |  |

Supplies for Sub-acromial shoulder and Knee joint injection

| 10 cc syringe | 22 or 25 G needle, 2 inches | Shorter-acting local anesthetic (~4cc)* | Longer-acting local anesthetic (~4cc) ^ | Injectable Steroid (40-80 mg for large joints) |
| --- | --- | --- | --- | --- |
| Ethyl chloride (Gebauer’s) spray | Betadine/Chlorhexidine | Gloves | Band-Aid | Gauze |

*Without epinephrine! ^only for bursa


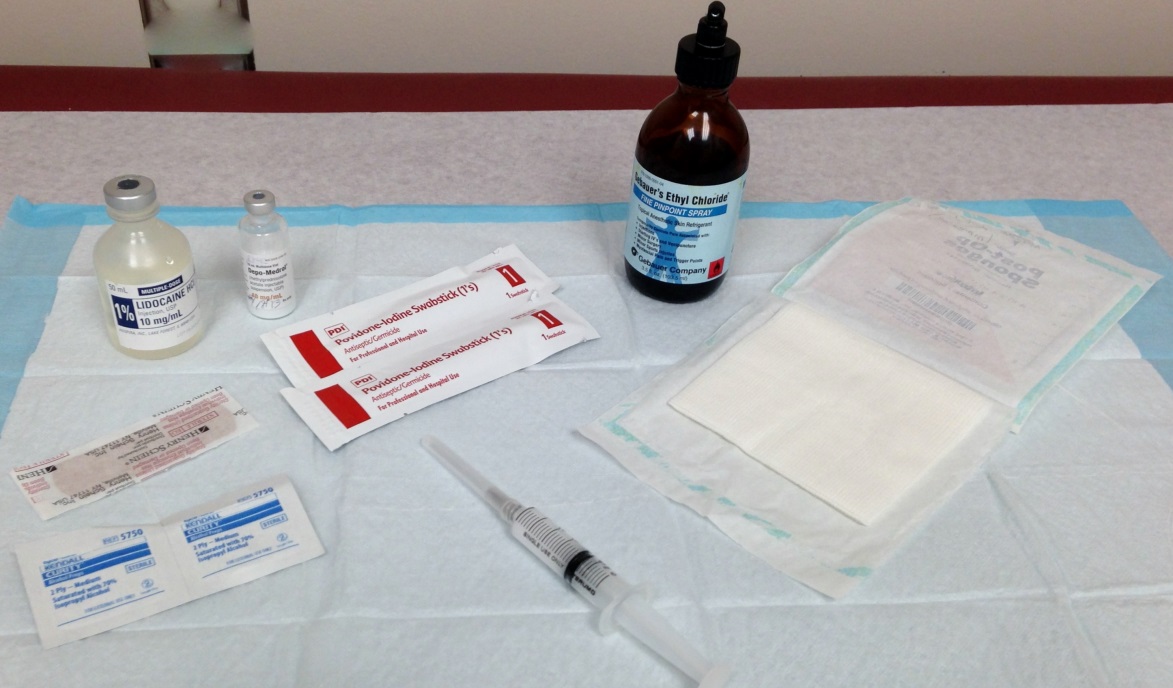


General Injection Tips

- Find your spot, mark with fingernail or pen tip
- Have all your supplies within arm’s reach of your dominant hand
- Shake the injection right before inserting the needle
- Put the needle through the skin quickly, pull it out of skin quickly
- You may feel a slight “give” once entering the capsule of joint or bursa
- The injection will flow in smoothly if you are in the right spot; if it doesn’t, reposition

Sub-acromial specific tips

- Find the acromion, then find the posterior lateral edge
- Your spot is 1 inch below, in the “soft” spot
- Point towards the coracoid when inserting
- Once in the skin, aim for the chin
- The needle is usually advanced to the hub, put depends on habitus

Knee specific tips

- The joint space between the femur and tibia/fibula is confluent the space behind the patella
- The needle usually does not need to be advanced to the hub
- For the lateral approach, the knee should be hanging off the bed at 90 degrees
  - Put the needle in the soft spot, lateral to the patellar tendon
  - Your needle should be parallel to the floor
  - Aim for the area just medial to the center point of the posterior portion of the knee
  - Small adjustments may be needed for severe OA with osteophytes
- For the superolateral approach, the patient should be supine with knee slightly flexed and supported by a rolled towel or pillow
  - The needle is placed lateral to the upper third of the patella
  - Push laterally and downward on the medial patella to open up the space
  - Aim superomedially for the top of the patella

What to warn your patient about after an injection

Increased pain for a 2-24 hours after injection, signs and symptoms of infection or significant bleeding
