# Supplementary material for: Improving Internal Medicine Resident Comfort With Shoulder and Knee Joint Injections Using an Injection Workshop
Source: MedEdPORTAL. 2020 Sep 28;16:10979. doi: 10.15766/mep_2374-8265.10979 (PMC7521064; doi:10.15766/mep_2374-8265.10979)
Supplement: Supplementary file 1 — Teaching Flow Plan.docxJoint Injections.pptxJoint Injection Handout.docxPreworkshop Questionnaire.docxPostworkshop Questionnaire.docxFour-Month Follow-Up Questionnaire.docx [file mep_2374-8265.10979-s001.zip › D. Preworkshop Questionnaire.docx]

**Joint Injection Precourse Questionnaire**

**Training Year**: PGY-1 PGY-2 PGY-3 PGY-4 PGY-5

**Degree**: M.D. D.O.

**Post-residency plans**: Primary Care Fellowship Hospitalist Other

Number of knee injections performed ***prior to*** starting residency:

0 1-5 6-10 11+

Number of sub-acromial shoulder injections performed ***prior to*** starting residency:

0 1-5 6-10 11+

Number of knee injections performed ***after*** starting residency:

0 1-5 6-10 11+

Number of sub-acromial shoulder injections performed ***after*** starting residency:

0 1-5 6-10 11+

I recognize the clinical indications for an intraarticular knee injection.

| 5 | 4 | 3 | 2 | 1 |
| --- | --- | --- | --- | --- |
| Very | More | Intermediate | Less | Not |
| Confident | Confident | Confidence | Confident | Confident |

I recognize the clinical indications for a subacromial shoulder injection.

| 5 | 4 | 3 | 2 | 1 |
| --- | --- | --- | --- | --- |
| Very | More | Intermediate | Less | Not |
| Confident | Confident | Confidence | Confident | Confident |

I can explain the risks and benefits of an intraarticular knee injection to my patients.

| 5 | 4 | 3 | 2 | 1 |
| --- | --- | --- | --- | --- |
| Very | More | Intermediate | Less | Not |
| Confident | Confident | Confidence | Confident | Confident |

(continued)

I can explain the risks and benefits of a subacromial shoulder injection to my patients.

| 5 | 4 | 3 | 2 | 1 |
| --- | --- | --- | --- | --- |
| Very | More | Intermediate | Less | Not |
| Confident | Confident | Confidence | Confident | Confident |

I know the supplies that I would need to perform an intraarticular knee injection.

| 5 | 4 | 3 | 2 | 1 |
| --- | --- | --- | --- | --- |
| Very | More | Intermediate | Less | Not |
| Confident | Confident | Confidence | Confident | Confident |

I know the supplies that I would need to perform a subacromial shoulder injection.

| 5 | 4 | 3 | 2 | 1 |
| --- | --- | --- | --- | --- |
| Very | More | Intermediate | Less | Not |
| Confident | Confident | Confidence | Confident | Confident |

I can perform an intraarticular knee injection safely and accurately.

| 5 | 4 | 3 | 2 | 1 |
| --- | --- | --- | --- | --- |
| Very | More | Intermediate | Less | Not |
| Confident | Confident | Confidence | Confident | Confident |

I can perform a subacromial shoulder injection safely and accurately.

| 5 | 4 | 3 | 2 | 1 |
| --- | --- | --- | --- | --- |
| Very | More | Intermediate | Less | Not |
| Confident | Confident | Confidence | Confident | Confident |
