# Supplementary material for: Improving Internal Medicine Resident Comfort With Shoulder and Knee Joint Injections Using an Injection Workshop
Source: MedEdPORTAL. 2020 Sep 28;16:10979. doi: 10.15766/mep_2374-8265.10979 (PMC7521064; doi:10.15766/mep_2374-8265.10979)
Supplement: Supplementary file 1 — Teaching Flow Plan.docxJoint Injections.pptxJoint Injection Handout.docxPreworkshop Questionnaire.docxPostworkshop Questionnaire.docxFour-Month Follow-Up Questionnaire.docx [file mep_2374-8265.10979-s001.zip › E. Postworkshop Questionnaire.docx]

**Joint Injection Postcourse Questionnaire**

Retrospective Self-Assessment

Prior to the teaching intervention, I recognized the clinical indications for an intraarticular knee injection.

| 5 | 4 | 3 | 2 | 1 |
| --- | --- | --- | --- | --- |
| Very | More | Intermediate | Less | Not |
| Confident | Confident | Confidence | Confident | Confident |

Prior to the teaching intervention, I recognized the clinical indications for a subacromial shoulder injection.

| 5 | 4 | 3 | 2 | 1 |
| --- | --- | --- | --- | --- |
| Very | More | Intermediate | Less | Not |
| Confident | Confident | Confidence | Confident | Confident |

Prior to the teaching intervention, I could explain the risks and benefits of an intraarticular knee injection to my patients.

| 5 | 4 | 3 | 2 | 1 |
| --- | --- | --- | --- | --- |
| Very | More | Intermediate | Less | Not |
| Confident | Confident | Confidence | Confident | Confident |

Prior to the teaching intervention, I could explain the risks and benefits of a subacromial shoulder injection to my patients.

| 5 | 4 | 3 | 2 | 1 |
| --- | --- | --- | --- | --- |
| Very | More | Intermediate | Less | Not |
| Confident | Confident | Confidence | Confident | Confident |

Prior to the teaching intervention, I knew the supplies that I would need to perform an intraarticular knee injection.

| 5 | 4 | 3 | 2 | 1 |
| --- | --- | --- | --- | --- |
| Very | More | Intermediate | Less | Not |
| Confident | Confident | Confidence | Confident | Confident |

Prior to the teaching intervention, I knew the supplies that I would need to perform a subacromial shoulder injection.

| 5 | 4 | 3 | 2 | 1 |
| --- | --- | --- | --- | --- |
| Very | More | Intermediate | Less | Not |
| Confident | Confident | Confidence | Confident | Confident |

(continued)

Prior to the teaching intervention, I was able to perform an intraarticular knee injection safely and accurately.

| 5 | 4 | 3 | 2 | 1 |
| --- | --- | --- | --- | --- |
| Very | More | Intermediate | Less | Not |
| Confident | Confident | Confidence | Confident | Confident |

Prior to the teaching intervention, I was able to perform a subacromial shoulder injection safely and accurately.

| 5 | 4 | 3 | 2 | 1 |
| --- | --- | --- | --- | --- |
| Very | More | Intermediate | Less | Not |
| Confident | Confident | Confidence | Confident | Confident |

Post-Course Self-Assessment

After the teaching intervention, I understand the indications for an intraarticular knee injection.

| 5 | 4 | 3 | 2 | 1 |
| --- | --- | --- | --- | --- |
| Very | More | Intermediate | Less | Not |
| Confident | Confident | Confidence | Confident | Confident |

After the teaching intervention, I understand the indications for a subacromial shoulder injection.

| 5 | 4 | 3 | 2 | 1 |
| --- | --- | --- | --- | --- |
| Very | More | Intermediate | Less | Not |
| Confident | Confident | Confidence | Confident | Confident |

After the teaching intervention, I can explain the risks and benefits of an intraarticular knee injection to my patients.

| 5 | 4 | 3 | 2 | 1 |
| --- | --- | --- | --- | --- |
| Very | More | Intermediate | Less | Not |
| Confident | Confident | Confidence | Confident | Confident |

(continued)

After the teaching intervention, I can explain the risks and benefits of a subacromial shoulder injection to my patients.

| 5 | 4 | 3 | 2 | 1 |
| --- | --- | --- | --- | --- |
| Very | More | Intermediate | Less | Not |
| Confident | Confident | Confidence | Confident | Confident |

(continued)

After the teaching intervention, I know the supplies that I would need to perform an intraarticular knee injection.

| 5 | 4 | 3 | 2 | 1 |
| --- | --- | --- | --- | --- |
| Very | More | Intermediate | Less | Not |
| Confident | Confident | Confidence | Confident | Confident |

After the teaching intervention, I know the supplies that I would need to perform a subacromial shoulder injection.

| 5 | 4 | 3 | 2 | 1 |
| --- | --- | --- | --- | --- |
| Very | More | Intermediate | Less | Not |
| Confident | Confident | Confidence | Confident | Confident |

After the teaching intervention, I am confident that I can perform an intraarticular knee injection safely and accurately.

| 5 | 4 | 3 | 2 | 1 |
| --- | --- | --- | --- | --- |
| Very | More | Intermediate | Less | Not |
| Confident | Confident | Confidence | Confident | Confident |

After the teaching intervention, I am confident that I can perform a subacromial shoulder injection safely and accurately.

| 5 | 4 | 3 | 2 | 1 |
| --- | --- | --- | --- | --- |
| Very | More | Intermediate | Less | Not |
| Confident | Confident | Confidence | Confident | Confident |

After the course, I am more likely to perform an intraarticular knee injection with my clinic patients if indicated.

| 5 | 4 | 3 | 2 | 1 |
| --- | --- | --- | --- | --- |
| Much more | Slightly more | No | Slightly less | Much less |
| Likely | Likely | Change | Likely | Likely |

After the course, I am more likely to perform a subacromial shoulder injection with my clinic patients if indicated.

| 5 | 4 | 3 | 2 | 1 |
| --- | --- | --- | --- | --- |
| Much more | Slightly more | No | Slightly less | Much less |
| Likely | Likely | Change | Likely | Likely |
